# Price competition and blockchain technology adoption strategies of agents on the digital platform

**DOI:** 10.3389/fpsyg.2022.984928

**Published:** 2022-10-14

**Authors:** Linfeng Wang, Guo Xie, Chen Chen

**Affiliations:** ^1^The Wang Yanan Institute for Studies in Economics, Xiamen University, Xiamen, China; ^2^School of Business Administration, Northeastern University, Shenyang, China; ^3^Horgos School of Business, Yili Normal University, Xinjiang, China

**Keywords:** digital platform, blockchain technology, price competition, consumer trust, agents

## Abstract

The rise of digital platforms intensifies the price competition among agents. Agents often use low price strategies to attract consumers. However, the low-price strategy is often filled with false information and consumers perceive the non-truthfulness of the price information. Then, consumers’ trust in agents gradually decreases, which inhibits the growth of online shopping. Blockchain is seen as a solution to the trust crisis between agents and consumers. Our research is based on two competing agents selling the same type of goods on the same platform. We discuss agents’ blockchain technology application strategies in three scenarios, which are defined by whether agents choose to apply blockchain technology to improve consumer trust. The results show that the application of blockchain technology is beneficial to agents only when consumer trust is low. Furthermore, the YN strategy is regarded as a possible equilibrium strategy, which depends on the blockchain application cost and consumer trust. Some extended cases are discussed for post-blockchain consumer welfare, cost-sharing contracts, dishonesty penalties, and variable blockchain costs, and the results show that the analysis in this manuscript is robust. Our findings have important practical significance for promoting the application of blockchain technology and alleviating the problem of price information asymmetry in platform shopping.

## Introduction

**Figure 1:**
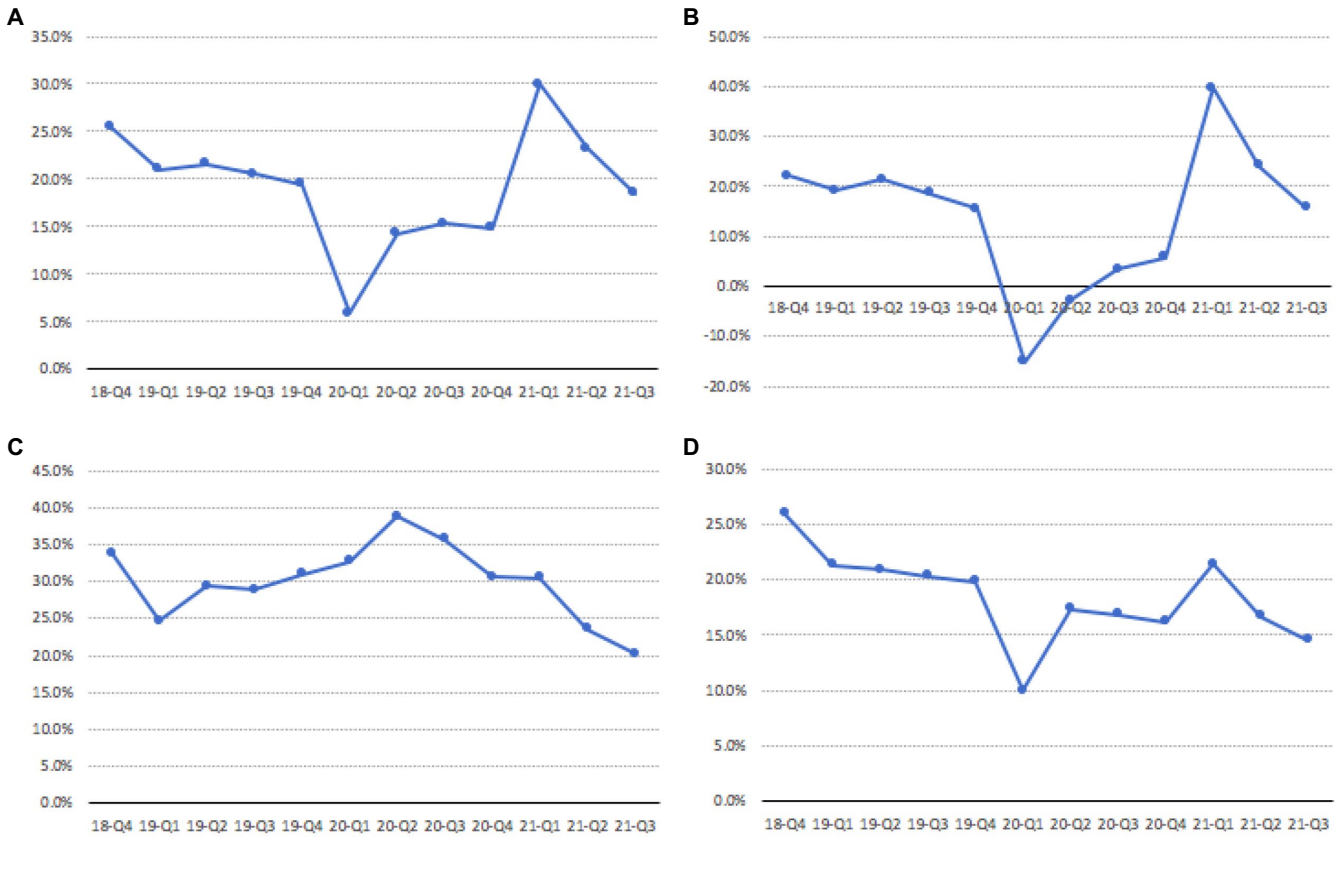
Growth rate of online shopping. **(A)** Total online retail sales, **(B)** Clothing, **(C)** Food and beverage, and **(D)** Daily necessities.

**Figure 2:**
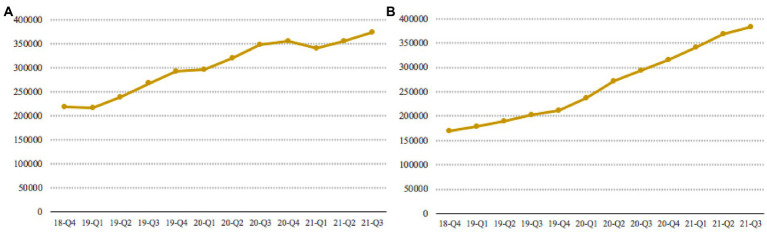
Growth in the number of agents on e-commerce platforms **(A)** Tmall and **(B)** JD.

With the rapid development of Internet technology, online shopping is playing an increasingly important role in people’s daily lives. After experiencing a period of rapid growth, online shopping is currently facing some obstacles and has fallen into a development dilemma (Liang et al., 2021). According to online shopping data from the National Bureau of Statistics of China, from the Q4 quarter of 2018 to the Q3 quarter of 2021 (China Internet Watch, 2021), the total growth rate of online shopping sales showed a significant downward trend (as shown in Figure 1A). The sales growth rate of clothing, food and daily necessities, which are popular items in online shopping, also decreased significantly (as shown in Figures 1B–D). On the contrary, the number of agents on the online shopping platform is on the rise. The growth trend of the number of agents of China’s major e-commerce platforms Tmall and JD is shown in Figure 2. It is easy to find that the number of agents has increased significantly in the past 3 years. It can be predicted that as the number of agents on the platform increases, the competition among agents will become more intense. In order to attract the attention of consumers, agents often use low-price strategies as a means of competition, but the strategy cannot be sustainable. In order to make up for the loss of revenue caused by the low-price strategy, agents have used a variety of improper tactics at different times to increase revenue. These tactics include fabricating the original price, using big data to discriminate pricing, and arbitrarily adjusting prices without fulfilling price commitments, etc. The improper tactics of the agents not only infringed on consumer welfare, but also caused consumers to feel injustice and disappointment, and gradually lose confidence in online shopping (Sun et al., 2020). A major problem is that in online shopping, it is difficult for consumers to verify the authenticity of an agent’s price information. Many consumers are attracted by false discounts, only to find out later that the usual selling price may be cheaper than the discounted price they purchased. Price competition among agents makes consumers lose confidence in online shopping, and online retail sales decline. So, can technical ways be used to alleviate the trust crisis between consumers and agents and promote the healthy development of online shopping?

Blockchain technology is developing rapidly at an undetected speed, realizing the linkage effect of multilateral industries and technologies, and improving the quality of development of the digital economy (Yli-Huumo et al., 2016). Blockchain is a new type of disintermediation database, which has the characteristics of unforgeable, open and transparent and collective maintenance, which can realize the information sharing of all parties on the chain. In the blockchain system, all transactions are time-tagged and verified by a consensus mechanism (An et al., 2020). Blockchain technology has been widely used in the fields of finance, energy, transportation, insurance and public management. The application of blockchain technology can establish a reliable cooperation mechanism to provide a solid trust between agents and consumers, thereby eliminating the lemon problem caused by the asymmetry of price information (Sikorski et al., 2017). Although blockchain technology can provide many benefits, the application of the technology comes at a cost. The application of blockchain technology requires the establishment of a system, content testing and regular maintenance, which will bring additional operating costs. The agents have the motivation to use blockchain technology, if and only if the benefits of using it are greater than the cost paid. So, what is the agent’s application strategy of blockchain technology? When will agents apply blockchain technology, and how will the application of blockchain technology affect market demand and consumer welfare? This manuscript discusses these issues by establishing blockchain technology application strategies for agents in different scenarios. Our research has important practical significance for advancing the application of blockchain technology and alleviating the problem of price information asymmetry between consumers and agents.

Our research is based on two competing agents selling exactly the same products, who are free to set up product information in their own stores on the platform. We deployed the blockchain technology application strategy for agents in three scenarios: (1) Neither agent applies blockchain technology (Strategy NN); (2) Only agent *i* applies blockchain technology (Strategy YN); (3) Both agents apply blockchain technology (Strategy YY). By comparing the amount of price information disclosure and the income of agents under the three strategies, we have obtained the equilibrium strategy of agent blockchain technology application. The application cost of blockchain and consumer trust are the key factors influencing the application of blockchain technology by agents. Finally, the case was extended to the consumer welfare, cost-sharing contracts, dishonest penalties and variable blockchain costs, showing that the results are relatively robust.

## Literature review

Our work mainly involves two research hotspots: the problem of price information asymmetry in online shopping and the application of blockchain technology. Many studies have explored the impact of price information asymmetry between merchants and consumers on economic efficiency. Krasker (1986) used the relationship between the number of new shares issued and the change of the company’s stock price to demonstrate that under the condition of information asymmetry, the company’s stock price is a decreasing function of the size of the issue, and information asymmetry will lead to underinvestment. Giesecke and Goldberg (2014) uses a structural model of corporate default risk to prove that information asymmetry can induce event premiums. Event premium refers to the sudden change in the price of securities when a company defaults. If public investors cannot observe the threshold asset value of the company’s management liquidation, they will face instantaneous default risk, which will also increase the credit premium and increase the company’s equity and debt financing costs. Hu et al. (2007) proposed a two-level supply chain model with asymmetric information. Retailers have a clearer understanding of customer demand distribution than manufacturers. Retailers are required by manufacturers to share demand forecast information. Retailer performance rises with distorted information. The manufacturer is aware of the retailer’s motives and doubts the authenticity of the information shared. The study proved that honest information sharing is impossible under a pure price contract, and retailers’ self-interested behavior weakens the cooperative relationship. Maeng and Choi (2016) studied the problem of limit pricing in the duopoly market under asymmetric demand status information. Equilibrium shows that when there is information asymmetry in the demand state, restricting pricing can prevent potential entrants from entering.

Some studies have also paid attention to how retailers can increase profits through asymmetry in price information, which is a hot topic. Tran and Desiraju (2017) pointed out that when consumers are sufficiently heterogeneous, group buying can help retailers implement price discrimination. Su and Geunes (2013) pointed out that when retailer demand is uncertain, suppliers can set price discounts at each stage within a limited planning period. This well-designed price promotion program can increase supplier profits. Emmanuel et al. (2013) used experimental methods to reveal that when the common value information of the auctioned items is incomplete, the average return of discriminatory auctions is higher than that of uniform price auctions. Colombo et al. (2021) investigated the duopoly model of companies inheriting asymmetric market shares and historical price discrimination. The results show that historical price discrimination makes the profit of the dominant company always lower than that of its competitors. The impact of information accuracy on industry profits is ambiguous. It also proves that the level of information accuracy has a non-monotonic effect on social welfare, which depends on the degree of asymmetry of inherited market shares. Sherman and Weiss (2012) found that when there is information asymmetry between consumers and firms, firms without adjacent competition exhibit forward and backward price rigidity. Melo (2007) pointed out that if regulators are biased against consumers or monopolistic producers, then in the case of asymmetric information, the equilibrium will be inefficient. But the impact on the equilibrium price and quality depends on the direction of the marginal effect of quality on the demand price. Different from their work, our research is based on two agents that sell homogeneous products and compete with each other on the platform. Agents publish inauthentic price information to gain a competitive advantage, at the expense of consumer trust. Agents will choose whether to apply blockchain technology to alleviate the crisis of trust caused by untrue price information. The purpose of our research is to enhance consumers’ confidence in online shopping and promote the sustainable development of e-commerce.

Blockchain is a distributed database that arranges data in chronological order and cannot be modified or forged. It has been widely used in the fields of finance, smart life and government. Many scholars have explored the application strategies and welfare effects of blockchain in various scenarios, and have harvested rich research results. Zhang et al. (2021) investigated the impact of blockchain technology on the strategic pricing of competing retailers, and the results show that it is not always advantageous for retailers to adopt blockchain technology in all situations. The two retailers will only adopt blockchain when consumers are less concerned about privacy and promote information transparency. Because the promotion of higher information transparency has increased consumers’ willingness to pay, exceeding the cost of consumers’ privacy concerns. Lu et al. (2021) used a questionnaire to study the application of blockchain technology in the elderly care industry (ECI). The research shows that corporate social responsibility, top management support, and organizational readiness have a positive impact on the willingness of companies to use blockchain. In addition, technological trust and information security positively affect the relative advantages of blockchain technology and indirectly affect the willingness to apply blockchain. Wang Y.Y. et al. (2021) pointed out that the application of blockchain energy in the energy system has limitations. The blockchain is not a panacea for the energy system. Because the component technology of the blockchain has its own common problems, the application of the energy blockchain should be accompanied by improvement measures that meet the actual needs of the energy system. Lu et al. (2021) investigated the blockchain adoption strategy of the operational decision-making module of the food supply chain consisting of a platform and a supplier. The conclusion points out that when both members of the supply chain adopt the blockchain, a win-win situation can be achieved. Sikorski et al. (2017) discussed the application of blockchain technology in the fourth industrial revolution (Industry 4.0). Studies have shown that blockchain technology has great development potential in improving the efficiency of the industrial revolution.

Some scholars have also evaluated the application effect of blockchain technology, and most of the experimental results believe that blockchain technology has a positive effect on promoting transaction trust. Christidis and Devetsikiotis (2016) investigate whether blockchain technology is applicable to the IoT field. They point out that deploying blockchain technology in IoT requires consideration of transaction privacy and the expected value of digital assets traded on the network. Although there are certain difficulties in the application of blockchain technology in the IoT area. But the combination of blockchain and IoT can trigger major changes in multiple industries, establishing new paths for business model innovation and distributed applications. Beck et al. (2016) developed a proof-of-concept prototype for solving the coffee shop payment trust problem. The results show that secure blockchain transactions can transform existing trust-based transaction systems. But scalability, cost, and volatility of traded currencies are obstacles. Khan and Byun (2021) proposed a peer-to-peer energy transaction and charging payment system for electric vehicles based on blockchain technology. The research shows that blockchain can alleviate trust and privacy issues and avoid the lack of transparency in the transaction process. Zhou et al. (2021) propose a decentralized reputation system (BC-DRS) in a blockchain-based e-commerce environment. BC-DRS is simulated in solid language on the popular blockchain platform, Ethereum. BC-DRS is simulated in solid language on the popular blockchain platform, Ethereum. Experimental results show that BC-DRS can protect product information and user reputation scores from intentional and unintentional modifications. It can effectively defend against common attacks such as unfair ratings and collusion. Our research is obviously different from the existing literature. (1) We apply blockchain technology to the scenario of asymmetric price information between agents and consumers on the platform. (2) We considered the blockchain application strategies of two competing agents in a variety of scenarios. (3) We have also expanded some influences that other external factors may have on blockchain applications.

In summary, the main contributions of our work are as follows. First, we have supplemented the research in the application field of blockchain technology. In order to solve the price information asymmetry between agents and consumers on the platform, we alleviate consumers’ concerns about false prices in online shopping by introducing blockchain technology. We have involved the comparison of blockchain applications in three scenarios. Such a multi-scenario comparative analysis can make our model closer to the reality. Secondly, we have identified that the cost of blockchain application and consumers’ trust in price information are key factors in the choice of agents’ strategies. The application cost of blockchain may inhibit its use in more areas. At the same time, there is a threshold for consumers’ trust in price information. In this threshold, the introduction of blockchain technology can improve consumer trust, and agents will get more profits, and vice versa. This result is less involved in the existing literature. Finally, our research scenario is based on the current anecdotal scenes of untrue prices in online shopping. Therefore, this study provides a new solution to the problem of price information asymmetry in online shopping.

## Models

### Deterministic mode

We consider an online shopping platform composed of two agents (*i* and *j*). The manufacturer produces a single product at a fixed unit cost and sells it through the agent’s platform channel. In addition, both agents can independently decide whether to introduce blockchain to improve the transparency of commodity prices. Therefore, there will be three different strategy structures.

1. Neither agent applies blockchain technology, which we call the NN strategy;
2. Only one of the two agents applies blockchain technology, which we call YN strategy;
3. Two agents simultaneously apply blockchain technology, which we call the YY strategy.

**Figure 3:**
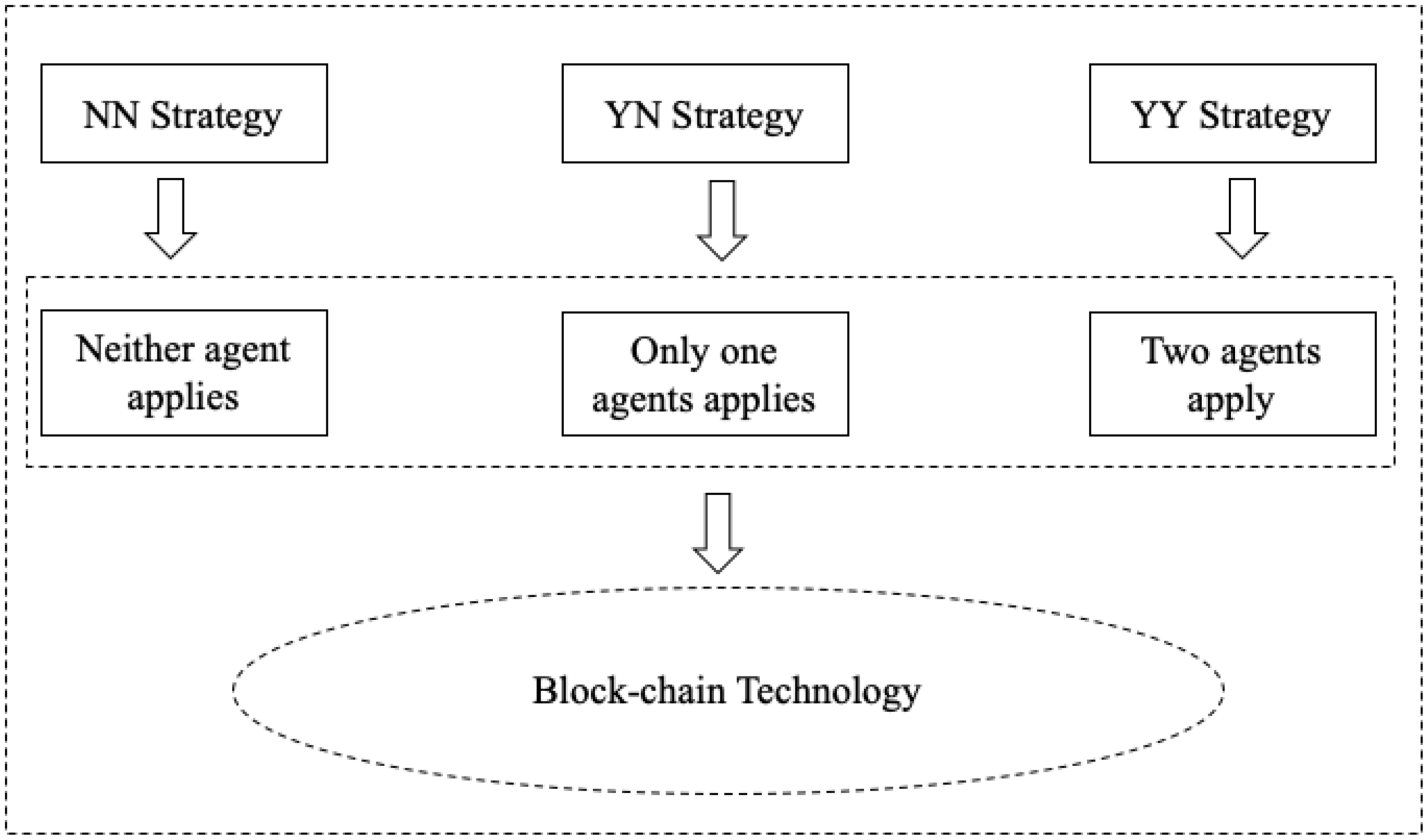
Strategy structure.

In order to facilitate analysis, we standardize the unit production cost to zero, which is a common practice in most literature. For the market structure, we set some useful signs to identify. We use w to represent the wholesale price between the manufacturer and the agent; $p_{\left\{i,j\right\}}$ represents the sales price of the product of agent *i*(*j*) on the platform. Each transaction of the agent on the platform needs to pay a certain fee rate to the platform provider, we use $c_{p}$ to represent the platform fee rate. We use $c_{B}$ to represent the unit cost of the agent applying blockchain technology. Our setting is similar to the current business model of the platform economy. In this manuscript, we first assume that the blockchain cost is exogenous, and then expand to the endogenous rate example in Section “Variable blockchain costs”. Table 1 summarizes the notation used. The timeline of the event is as follows. First, the agent’s choice of blockchain technology application has resulted in three strategic structures. Secondly, agents make pricing decisions at the same time based on three strategic structures. We assume that the blockchain application cost and market demand are complete information, and agents aim to maximize profits (Wang L. et al., 2021). The strategic structure is shown in Figure 3.

**Table 1:** Notations and descriptions.

Notation	Description
α	Base market
*W*	Wholesale prices
$p_{\left\{i,j\right\}}$	Product sales price
$\theta_{i,j}$	Disclosure of agent price information
β	Consumer sensitivity to cross-price
μ	Consumer sensitivity to cross-price disclosure
V	Consumer valuation of purchased goods
τ	Consumers’ trust in the agent’s price information
$c_{p}$	The unit rate charged by the platform to the agent
$c_{B}$	Unit fixed costs for agents to Make blockchain Investments
*F*	Dishonest punishment
φ	Probability of dishonest behavior being caught
$c_{vB}$	Variable unit blockchain cost
η	Blockchain cost sharing ratio

#### Utility function

In order to improve consumer trust to promote increased demand, agents are motivated to use technological ways to improve consumer fears of false prices. The utility function obtained by consumers purchasing a product can be expressed as:


(1) $$U=V_{\left\{i,j\right\}}-\left(\theta_{T}-\theta_{i}-\theta_{j}\right)-\beta p+\tau \left(\theta_{i}-\mu \theta_{j}\right)$$

This utility function has been used many times for products in multiple market segments and allows to reflect the situation of each segment (Bruno and Alessandro, 2018; Dan et al., 2020; Wang Y.Y. et al., 2021). Among them, V is the consumer’s valuation of the purchased goods, and the subscripts *i* and *j* denote different agents. $\theta_{T}$ represents the complete transparency of the agent’s price, which is the complete disclosure of the price information of all products of the agent. $\theta_{i}$ represents the price disclosure of agent *i*. $\theta_{j}$ represents the price disclosure of agent *j*. p represents the selling price of the product. β represents the consumer’s sensitivity to cross-price. *μ* indicates that consumers are sensitive to the disclosure of cross-price information. It is easy to understand that consumers will not only be affected by the current pricing of competitors, but also be affected by the disclosure of historical price information of competitors. We denote τas the consumer’s degree of trust in the agent’s price information (0≤τ<1). When τ=0, it means that consumers do not trust price information at all, which is common on some online shopping platforms that boast about product price reductions and false original prices. When τ=1, it means that consumers completely trust the price information, which can be met when the agent implements price information authentication for all products.

### Demand function

We assume that there is a deterministic underlying market for products, and consumers are sensitive to price fluctuations of commodities. Consumer demand for agent *i* decreases as its own price increases and its competitor’s price decreases, and vice versa. Each agent can freely decide to join the blockchain technology or maintain the existing way of price management. The agent needs to decide the strategy choice, and at the same time formulate the corresponding commodity price (*p*) and the publication level of price information (*θ*). The superscripts represent different scenarios, and the demand function can be expressed as follows:

1. In the NN scenario, both agents do not use blockchain technology, and the demand function can be expressed as:
(2) $$D_{i}^{\mathrm{NN}}=\alpha -p_{i}+\beta p_{j}+\tau \left(\theta_{i}-\mu \theta_{j}\right)$$
(3) $$D_{j}^{\mathrm{NN}}=\alpha -p_{j}+\beta p_{i}+\tau \left(\theta_{j}-\mu \theta_{i}\right)$$
  Formulas (2), (3) represent the market demand for the products of the two agents. To be consistent with reality, we assume that consumers are more sensitive to current prices than historical prices.
2. In the YN scenario, only one agent adopts blockchain technology, we set agent *i* to adopt blockchain technology, and agent *j* does not. Due to the application of blockchain technology, the price information of agent *i* is verifiable. Consumers completely trust the price of agent *i*. However, consumers still have doubts about the price of agent *j*. Therefore, the demand function is expressed as:
(4) $$D_{i}^{\mathrm{YN}}=\alpha -p_{i}+\beta p_{j}+\theta_{i}-\tau \mu \theta_{j}$$
(5) $$D_{j}^{\mathrm{YN}}=\alpha -p_{j}+\beta p_{i}+\tau \left(\theta_{j}-\mu \theta_{i}\right)$$
3. In the YY scenario, both agents apply blockchain technology, and the price information disclosed by both parties can be fully authenticated. Therefore, consumers fully trust the price information, and the demand function is expressed as:
(6) $$D_{i}^{\mathrm{YY}}=\alpha -p_{i}+\beta p_{j}+\theta_{i}-\mu \theta_{j}$$
  (7) $$D_{j}^{\mathrm{YY}}=\alpha -p_{j}+\beta p_{i}+\theta_{j}-\mu \theta_{i}$$

In order to capture the attention of consumers, agents will disclose positive information about product prices to consumers, including publishing more price discount information, industry price information and historical price information comparison, to create a “cheapest today” situation. This information can stimulate consumers to be more sensitive to product prices and encourage consumers to buy. The premise is that the information needs to be less publicly available previously. Although the products sold by the agents are not updated, this newly disclosed information can potentially increase the attractiveness of the products. Consistent with the reality, we need to emphasize that there is a cost in the amount of newly announced price information, and there is a positive correlation between the cost and the amount of price information disclosure (Wang F. et al., 2021). We use the coefficient t to represent the cost of new price information disclosure. Therefore, the cost of price information disclosure is defined as:

(8) $$\frac{1}{2}t\theta_{i}{}^{2}$$

However, since the published price information has not been filtered and authenticated, consumers do not fully trust the price information published by the agent. In order to alleviate consumers’ doubts about price information, agents can apply blockchain technology for information authentication and real-time updates. The initial setup cost of blockchain technology is huge and is often seen as a silent cost. We use the coefficient $c_{B}$ to represent the blockchain unit fixed cost. We refer to common practice and exclude variable costs in the base model, but we still need to consider the noise impact of fixed costs in the stability analysis. The reason is that the number of information nodes and ports that need to be added increases in the same direction as the information application cost, which indicates that the application cost of blockchain technology and the amount of price information disclosure increase at the same time. In order to avoid the shock wave effect on the results, we assume that the blockchain fixed cost coefficient is relatively large (Chen et al., 2018).

## Model analysis

We use a backward-order solution to find possible solutions in various scenarios. According to the general concept, the profit function of agents in various scenarios is quasi-concave, and the marginal profit decreases with the increase of sales volume (Allcott and Sweeney, 2017). According to the profit maximization, the agent’s optimal selling price, optimal information disclosure amount and consumer demand can be obtained.

### Strategy-[NN]

In the NN scenario, both agents do not use blockchain technology, and consumers have doubts about price information. The optimal profit function of agent *i*(*j*) can be expressed as:


(9) $$\pi_{i}^{\mathrm{NN}}=\left(p_{i}-w-c_{p}\right).D_{i}^{\mathrm{NN}}-\frac{1}{2}t\theta_{i}{}^{2}$$

(10) $$\pi_{j}^{\mathrm{NN}}=\left(p_{j}-w-c_{p}\right).D_{j}^{\mathrm{NN}}-\frac{1}{2}t\theta_{j}{}^{2}$$

We substitute the demand function Equations (1), (3) in the NN scenario into Equations (9), (10) to obtain the relevant equilibrium solutions for the optimal sales price, sales quantity and information disclosure.

*Proposition 1:* For the NN scenario, the optimal sales price and price information disclosure amount of agent *i*(*j*) are:

$$p_{i}^{\mathrm{NN}}=p_{j}^{\mathrm{NN}}=\frac{t\alpha +\left(t+\left(\mu -1\right)\tau^{2}\right)\left(w+c_{p}\right)}{t\left(2-\beta \right)+\left(\mu -1\right)\tau^{2}}$$

$$\theta_{i}^{\mathrm{NN}}=\theta_{j}^{\mathrm{NN}}=\frac{\tau \left(\alpha +\left(\beta -1\right)\left(w+c_{p}\right)\right)}{t\left(2-\beta \right)+\left(\mu -1\right)\tau^{2}}$$

Substituting the optimal sales price and the amount of price information disclosure into formula (9), (10), we can obtain the equilibrium profit solution of the two agents:


$$\pi_{i}^{\mathrm{NN}}=\pi_{j}^{\mathrm{NN}}=\frac{t\left(2t-\tau^{2}\right){\left(\alpha +\left(\beta -1\right)\left(w+c_{p}\right)\right)}^{2}}{2\left(t\left(2-\beta \right)\right)+{\left(\left(\mu -1\right)\tau^{2}\right)}^{2}}$$

From Proposition 1, it can be found that the optimal selling price, the amount of price information disclosure and the maximum profit have a great relationship with the trust degree of public information.

*Corollary 1:* For the NN scenario, we have the following:

1. $\frac{dD_{\left\{i,j\right\}}}{d\tau }>0$; $\frac{dp_{\left\{i,j\right\}}^{\mathrm{NN}}}{d\tau }>0$; $\frac{d\theta_{\left\{i,j\right\}}}{d\tau }>0$;
2. if $\tau <\tau^{*}$, $\frac{d\pi_{\left\{i,j\right\}}^{\mathrm{NN}}}{d\tau }>0$; Otherwise, $\frac{d\pi_{\left\{i,j\right\}}^{\mathrm{NN}}}{d\tau }<0$;

From Corollary 1(1), it can be found that agents increase the trust in the price information of the product leading to a joint increase in the demand for the product and the release of price information. Although commodity prices will also rise, it is still profitable for agents. The reason is that the increased transparency of price information by agents can gain consumers’ trust and stimulate more purchases (Akhtaruzzaman et al., 2020). With high consumer trust, agents are motivated to disclose more price information. There is also an opposite phenomenon. If consumers are highly suspicious of the price information released by the agent, the agent should reduce the amount of price information disclosed. Consistent with the reality, many agents do not explain changes in commodity price information (Bai and Sarkis, 2020).

From (2) of Inference 1, we find that there is a threshold for consumers’ trust in price information. The agents have an incentive to disclose information if and only if the agent’s marginal revenue is positive. Specifically, the publication of information has costs, and the publication of some information may bring negative effects to agents (for example: in real life, exaggerated price advertising information may make consumers more suspicious). When the consumer’s price trust in the agent is lower than the threshold, it is profitable to increase the price information authentication to improve the transparency of the information (Zhang et al., 2021). When consumers’ trust in price information exceeds the threshold, the increased amount of information disclosure cannot cover the cost, and the situation for agents will get worse. It shows that agents are not always motivated to increase the trust of price information.

### Strategy-[YN]

In the YN scenario, agent *i* applies blockchain technology to disclose commodity price information. Any consumer can freely verify the authenticity of commodity price information, and consumers fully trust the information published by agent *i*. However, agent *j* has not applied blockchain technology, and the price information is still unverifiable, and consumers are dubious.

Without loss of generality, we assume that agent *i* applies blockchain technology and agent *j* does not. Therefore, the profits of the two agents can be listed separately as follows:


(11) $$\pi_{i}^{\mathrm{YN}}=\left(p_{i}-w-c_{p}-c_{B}\right).D_{i}^{\mathrm{YN}}-\frac{1}{2}t\theta_{i}{}^{2}\;$$

(12) $$\pi_{j}^{\mathrm{YN}}=\left(p_{j}-w-c_{p}\right).D_{j}^{\mathrm{YN}}-\frac{1}{2}t\theta_{j}{}^{2}$$

We substitute the demand function Equations (4), (5) in the YN scenario into Equations (11), (12) to obtain the relevant equilibrium solutions of the optimal sales price, sales quantity and price information disclosure:

*Proposition 2:* For the YN scenario, the optimal sales price of agent *i*(*j*) and the corresponding optimal price information disclosure are:

$$\begin{array}{c} p_{i}^{\mathrm{YN}}=\frac{w+c_{p}+\alpha }{\left(2-\beta \right)}+\frac{U1}{A\left(\beta^{2}-4\right)},\\ p_{j}^{\mathrm{YN}}=\frac{w+c_{p}+\alpha }{\left(2-\beta \right)}+\frac{U2}{A\left(\beta^{2}-4\right)} \end{array}$$

$$\begin{array}{c} \theta_{i}^{\mathrm{YN}}=\frac{\left(\alpha +\left(w+c_{p}\right)\left(\beta -1\right)\right)B+Fc_{B}}{A},\\ \theta_{j}^{\mathrm{YN}}=\frac{\left(\alpha +\left(w+c_{p}\right)\left(\beta -1\right)\right)G+Hc_{B}}{A} \end{array}$$

Substituting the optimal sales price and the amount of price information disclosure into formula (11), (12), we can obtain the equilibrium profit solution of the two agents:


$$\pi_{i}^{\mathrm{YN}}=\frac{t\left(2t-1\right){\left(\begin{array}{l} \left(\alpha +\left(w+c_{p}\right)\left(\beta -1\right)\right)\left(\beta^{2}-4\right)\\ \left(t\left(2+\beta \right)-\left(1+\mu \right)\tau^{2}\right)+c_{B}\left(\beta^{2}-4\right)\\ \left(t\left(\beta^{2}-2\right)+\left(1-\beta \mu \right)\tau^{2}\right) \end{array}\right)}^{2}}{2A^{2}{\left(\beta^{2}-4\right)}^{2}}$$

$$\pi_{j}^{\mathrm{YN}}=\frac{t\left(2t-\tau^{2}\right){\left(\begin{array}{l} \left(\alpha +\left(w+c_{p}\right)\left(\beta -1\right)\right)\left(\beta^{2}-4\right)\\ \left(1-t\left(2+\beta \right)+\mu \tau \right)+c_{B}\left(\beta^{2}-4\right)\\ \left(\left(t-1\right)\beta +\mu \tau \right) \end{array}\right)}^{2}}{2A^{2}{\left(\beta^{2}-4\right)}^{2}}$$

Due to the existence of market competition, the decision of agent *i* will affect the changes of the overall market. After the agent *i* applies the blockchain technology, the commodity price, the amount of price information disclosure, consumer trust and market demand will also change accordingly. However, even though agent *j*’s strategy has not changed, it will still be implicated. Consumers with more price information may switch the purchasing channel to agency *i*. Cost-sensitive consumers may switch to agent *j*. This means that even though only one agent applies blockchain technology, competitors in the same market will be affected by the association.

*Corollary 2:* For the YN scenario, we have the following:

Part I, the impact of consumer trust:

1. In terms of consumer trust in public price information, if and only if βμ≥1, $\;\frac{d\theta_{i}^{\mathrm{YN}}}{d\tau }>0,\mathrm{Otherwise},\frac{d\theta_{i}^{\mathrm{YN}}}{d\tau }<0;\mathrm{if}\;t<\beta$, $\;\frac{d\theta_{j}^{\mathrm{YN}}}{d\tau }>0,\mathrm{Otherwise},\frac{d\theta_{j}^{\mathrm{YN}}}{d\tau }<0.$
2. if and only if β ≥ 2*μ*, Consumers are more price sensitive than information, $\;\frac{dp_{i}^{\mathrm{YN}}}{d\tau }>0,\frac{dp_{j}^{\mathrm{YN}}}{d\tau }>0$,$\;\frac{d\pi_{i}}{d\tau }>0;$ $\mathrm{Otherwise,}\frac{dp_{i}^{\mathrm{YN}}}{d\tau }<0$;$\;\frac{dp_{j}^{\mathrm{YN}}}{d\tau }<0$;$\;\frac{d\pi_{i}^{\mathrm{YN}}}{d\tau }<0;$In addition, the case of agent *j* is $\frac{d\pi_{j}^{\mathrm{YN}}}{d\tau }>0$.

In Inference 2(1), it can be found that consumers’ trust in public prices is affected by cross-price and cross-information disclosure. If and only if (*βμ* ≥ 1), the increase of price information disclosure by agent *i* can promote consumers’ trust, and vice versa. However, the situation of agent *j* is different, if and only if (*t* < *β*), that is, consumers are more sensitive to cross-price information than the cost of new price information disclosure, and the increase of price information disclosure by agent *j* will help improve consumer trust.

In Corollary 2(2), it can be found that the situation of agent *i* depends on the sensitivity of consumers to the disclosure of price information. If and only if the consumer’s sensitivity to price exceeds the disclosure of price information (*β* > 2 *μ*), it is beneficial for agent *i* to use blockchain technology to authenticate and disclose information to improve consumer sensitivity. The reason is that improving consumers’ perception of information can stimulate consumers to increase their demand. When consumers are more sensitive to price information disclosure than price (*β* < 2 *μ*), agent *i* has no incentive to provide more information. Since consumers are too sensitive to price information, an increase in price information will have a greater impact on consumers (Liu et al., 2021). However, for agent *j*, there is always an incentive to provide more information when consumers are less sensitive to price disclosure information. The reason is that providing more price disclosure information attracts consumers and its cost of price disclosure information is lower than that of agent *i*. In general, in the YN scenario, both agents will actively improve consumers’ information sensitivity when consumers are less aware of price disclosure information. The agent *i* that applies the blockchain will be affected by the consumer’s trust in the price information of the agent *j* that does not apply the blockchain. In addition, when consumers have a high degree of perception of current prices, only agents who do not apply blockchain have the incentive to increase consumers’ sensitivity to price disclosure (Hmk et al., 2021). Because in this scenario, the agent *j* that does not apply the blockchain can publish more price information to attract the attention of consumers.

Part II, the impact of blockchain application costs:

1. When βμ≥1, then$\;\frac{d\theta_{i}^{\mathrm{YN}}}{dc_{B}}>0;\frac{dD_{i}^{\mathrm{YN}}}{dc_{B}}>0;\mathrm{Otherwise},\frac{d\theta_{i}^{\mathrm{YN}}}{dc_{B}}<0;\frac{dD_{i}^{\mathrm{YN}}}{dc_{B}}<0$; For agent *i*, the price change is $\frac{dp_{i}^{\mathrm{YN}}}{dc_{B}}>0$.
2. When$\begin{array}{l} \;\beta >\mu ,\mathrm{then}\;\frac{d\theta_{j}^{\mathrm{YN}}}{dc_{B}}>0;\frac{dp_{j}^{\mathrm{YN}}}{dc_{B}}>;0\frac{dD_{j}^{\mathrm{YN}}}{dc_{B}}>0;\\ \mathrm{Otherwise},\frac{d\theta_{j}^{\mathrm{YN}}}{dc_{B}}<0;\frac{dp_{j}^{\mathrm{YN}}}{dc_{B}}<0 \end{array}$; $\;\frac{dD_{j}^{YN}}{dc_{B}}<0$.
3. When $\beta >1,\mathrm{then}\frac{d\pi_{i}^{\mathrm{YN}}}{dc_{B}}>0;\mathrm{Otherwise},\frac{d\pi_{i}^{\mathrm{YN}}}{dc_{B}}<0$. For agent *j*, its profit is also related to the cost of blockchain application of competitors, if2t>τ, $\mathrm{then}\frac{d\pi_{j}^{\mathrm{YN}}}{dc_{B}}$>0; Otherwise, $\frac{d\pi_{j}^{\mathrm{YN}}}{dc_{B}}$<0.

As shown in Corollary 2(1), after agent *i* applies blockchain technology, in addition to being affected by the cost of blockchain, the amount of price information disclosure and demand also depends on the sensitivity of consumers to cross-price and cross-price information disclosure. If and only ifβμ≥1, the price information disclosure and demand of agent *i* will increase together with the blockchain cost, and vice versa. The product sales price of agent *i* also rises together with the cost of the blockchain. That is, the application of blockchain technology makes the selling price of agent *i*’s products rise. The situation for agent *j* is more complicated. Specifically, if and only if β<μτ, the amount of information disclosure, product sales price and demand and the cost of the blockchain increase together. Under the influence of blockchain costs, the profit performance of the two agents is also different. When consumers are more sensitive to cross-pricing (*β* > 1), agent *i* is able to make more profit by applying blockchain technology, and vice versa, it is worse off. For agent *j*, it is able to gain more profit when the information disclosure cost is greater than the consumer trust (2t>τ).

It is worth mentioning that even though agent *j* does not apply blockchain technology, the sales price of agent *j* will also increase due to the influence of agent *i* who applies blockchain technology. The reason is that on the same platform, agent *j* will be influenced by competitors applying blockchain. Agents *j* that do not use blockchain technology will increase the amount of price information disclosed, which is profitable despite the increased cost. Since the cost of disclosing the price information of agent *i* using blockchain technology is relatively high, the situation of agent *j* is just the opposite. So agent *j* always has an incentive to capture consumers by publishing more information. In general, in the YN scenario, the cost of blockchain will have a detrimental effect on agents who apply the technology, and a promotion effect on platforms that do not apply the technology.

*Lemma 1:* Comparison of blockchain adoption strategies in NN and YN scenarios:

1. For agent *i*, the disclosure of price information is $\theta_{i}^{\mathrm{YN}}>\theta_{i}^{\mathrm{NN}};$ The disclosure of price information for agent *j* is when $t\leq \frac{1}{2}$, $\mathrm{then}\;\theta_{j}^{\mathrm{YN}}>\theta_{j}^{\mathrm{NN}}$; Otherwise, $\theta_{j}^{\mathrm{YN}}<\theta_{j}^{\mathrm{NN}}$; The comparison of the disclosure of the price information of the two agents is when t>τ, $\theta_{i}^{\mathrm{YN}}>\theta_{j}^{\mathrm{YN}}$; Otherwise, $\theta_{i}^{\mathrm{YN}}<\theta_{j}^{\mathrm{YN}}$.
2. There are two thresholds for blockchain application costs$\;c_{1}^{a}\;\mathrm{and}\;c_{1}^{b}$:if $0\leq c_{B}\langle c_{1}^{a},\pi_{i}^{\mathrm{YN}}\rangle \pi_{\left\{i,j\right\}}^{\mathrm{NN}};$ Otherwise $c_{1}^{a}<c_{B}<c_{1}^{b}$,$\;\pi_{i}^{\mathrm{YN}}<\pi_{\left\{i,j\right\}}^{\mathrm{NN}}$.
3. There are two thresholds for blockchain application costs $c_{1}^{c}\;\mathrm{and}\;c_{1}^{d}:\mathrm{if}\;0\leq c_{B}\langle c_{1}^{c},\pi_{j}^{\mathrm{YN}}\rangle \pi_{\left\{i,j\right\}}^{\mathrm{NN}};$ Otherwise if $c_{1}^{c}<c_{B}<c_{1}^{d}$, $\;\pi_{j}^{\mathrm{YN}}<\pi_{\left\{i,j\right\}}^{\mathrm{NN}}.$
4. The blockchain application cost has a threshold$\;c_{1}^{e}:\mathrm{if}\;0\leq c_{B}\langle c_{1}^{e},\pi_{i}^{\mathrm{YN}}\rangle \pi_{j}^{\mathrm{YN}};$ Otherwise $c_{1}^{e}\leq c_{B}<c_{1}^{b}$, $\pi_{i}^{\mathrm{YN}}<\pi_{j}^{\mathrm{YN}}$.

The expressions for $c_{1}^{a}$, $c_{1}^{b},c_{1}^{c},c_{1}^{d}\;\mathrm{and}\;c_{1}^{e}$ are shown in the Appendix.

From Lemma 1(1), it can be found that compared with the NN scenario, the agent *i* will disclose more price information in the YN scenario. Since consumers completely trust its information after adopting blockchain technology, consumers can be attracted by publishing new information. For agent *j*, the impact caused by blockchain still needs to be considered despite the fact that blockchain technology is not applied (Choi et al., 2020). Agent *j* has an incentive to disclose more price information when the coefficient of the cost of additional information disclosure is low $\left(t\leq \frac{1}{2}\right)$. Otherwise, when the cost of information is high, it is not a good idea for agent *j* to disclose more information because consumers are suspicious of the released information. In addition, we compare the amount of price information disclosed by the two agents in the YN scenario. It can be found that agent *i* discloses more price information than agent *j* when the cost of additional information disclosure is greater than consumers’ trust (t>τ). The reason is that in the YN scenario, the price information in the market is full of noise, and both real information and false information exist. Although there is a cost in the amount of newly disclosed information, as long as the cost is greater than the consumer’s trust, the agent *i* can increase consumer trust by publishing more real information, thereby gaining more market share.

From Lemma 1(2), it can be found that when the blockchain application cost is lower than the threshold ($c_{1}^{a}$), it is very beneficial to the agent *i*, and its profit will increase. The reason is that the application of blockchain technology alleviates consumer concerns about price gouging. Although there will be additional costs, the application of blockchain brings more demand to agent *i*, which can hedge against the cost of new technology application. However, as the cost of blockchain application rises, the new profit cannot cover the cost, and the situation of agent *i* will get worse. For agent *j*, the situation will be even more different. When the cost of blockchain application is low, the agent *j* who ignores the blockchain technology will suffer losses (see Lemma 1(4)). Since the blockchain cost is low, the agent *i* applying the blockchain is fully trusted by the consumers, thus occupying the majority of the market share. Moreover, the profit of agent *i* applying blockchain technology decreases while the profit of agent *j* becomes better when the blockchain cost exceeds the threshold ($c_{1}^{e}$; see Citation 1(4)). Because of the rising cost of blockchain, the positive effects of applying blockchain outweigh the negative effects of rising costs (Basu et al., 2019). Agents *j* that do not apply blockchain technology have cost advantages and can accommodate more demand shifts from price-sensitive consumers. From Lemma 1(2), it can be found that when the blockchain application cost is low, it is very beneficial to the agent *i* applying the blockchain technology, and promotes it to publish more real price information. When the blockchain cost is at an intermediate threshold, such as $c_{1}^{a}\leq c_{B}<c_{1}^{b}\;\mathrm{and}\;c_{1}^{b}\leq c_{B}<c_{1}^{e}$, it may cause losses to both agents. There are more unauthenticated price information on the platform than authenticated ones (see Lemma 1(1) and (3)). An agent *i* applying blockchain technology will suffer more losses than an unapplied one. The final result is that no one wants to apply blockchain technology. However, it is interesting to find that when $c_{B}\in \left\{c_{1}^{a},c_{1}^{c},c_{1}^{e}\right\}$, the agent *i* who applied the blockchain technology gained a positive profit, and the agent *j* who did not apply the blockchain technology also better than before, indicating that there is a positive network externality in the application of blockchain technology.

Comparing the profit of NN strategy and YN strategy, it can be found that when the application cost of blockchain technology is low, the agent *i* who applies blockchain can get more profit and agent *j* who does not apply blockchain technology will have less profit. However, when the application cost of blockchain technology is high, it reduces the profit of the agent *i* who applies it, and makes the situation of the agent *j* who ignores it better. When the cost of blockchain technology application is at an intermediate threshold, blockchain technology may make the situation better for both agencies.

### Strategy-[YY]

In the YY scenario, both agents apply blockchain technology. Consumers can verify and identify the authenticity of the price information of both agents, and the price information on the market is all real and valid. The application of blockchain increases the cost of the agent, and the profit of the agent is expressed as follows:


(13) $$\pi_{i}^{\mathrm{YY}}=\left(p_{i}-w-c_{p}-c_{B}\right).D_{i}^{\mathrm{NN}}-\frac{1}{2}t\theta_{i}{}^{2}\;$$

(14) $$\pi_{j}^{\mathrm{YY}}=\left(p_{j}-w-c_{p}-c_{B}\right).D_{j}^{\mathrm{NN}}-\frac{1}{2}t\theta_{j}{}^{2}\;$$

We substitute the demand function Equations (6), (7) in the YN scenario into Equations (13), (14) to obtain the relevant equilibrium solutions of the optimal sales price, sales quantity and price information disclosure amount:

*Proposition 3:* For the YY scenario, the optimal sales price of agent *i*(*j*) and the corresponding optimal price information disclosure are:

$$p_{i}^{\mathrm{YY}}=p_{j}^{\mathrm{YY}}=\frac{t\alpha -\left(w+c_{p}+c_{B}\right)\left(\mu -1-t\right)}{1-\left(2-\beta \right)t-\mu }$$

$$\theta_{i}{}^{\mathrm{YY}}=\theta_{j}{}^{\mathrm{YY}}=\frac{\left(w+c_{p}+c_{B}\right)\left(1-\beta \right)-\alpha }{1+\left(2-\beta \right)t-\mu }$$

Substituting the optimal sales price and the amount of price information disclosure into formula (13), (14), we can obtain the equilibrium profit solution of the two agents:


$$\pi_{i}^{\mathrm{YY}}=\pi_{j}^{\mathrm{YY}}=\frac{{\left(\alpha +\left(w+c_{p}+c_{B}\right)\left(\beta -1\right)\right)}^{2}t^{3}\left(2-\beta \right)A-t\left(1-\mu \right)B}{2{\left({\left(\mu -1\right)}^{2}-t^{2}{\left(\beta -2\right)}^{2}\right)}^{2}}$$

It is worth mentioning that in the YY scenario, consumers have complete trust in the agent’s price information (τ=1). The equilibrium solution is only related to the cost of blockchain application. From Proposition 3, we get the following Corollary 3:

*Corollary 3:* When $\beta \langle 1,\frac{d\theta_{\left\{i,j\right\}}^{\mathrm{YY}}}{dc_{B}}\rangle 0;\mathrm{Otherwise},\frac{d\theta_{\left\{i,j\right\}}^{\mathrm{YY}}}{dc_{B}}<0;$ The change in consumer demand is $\frac{dD_{i,j}^{\mathrm{YY}}}{dc_{B}}<0$; The change in price and profit for both agents is $\frac{dp_{\left\{i,j\right\}}^{\mathrm{YY}}}{dc_{B}}>0\;\mathrm{and}\;\frac{d\pi_{\left\{i,j\right\}}^{\mathrm{YY}}}{dc_{B}}<0$.

When both agents apply blockchain technology, the agent’s strategy choice is no longer the same as in the YN scenario, especially for the agent *i* who applies blockchain technology in the YN scenario. Specifically, in the YY scenario, the product prices of both agents have risen due to the application of blockchain technology. However, in the YN scenario, the agent’s pricing is also determined by the competitor’s price and the consumer’s sensitivity to price information. When agent *j* also applies blockchain technology, the disclosure of price information depends on the cost of blockchain. Both agents tend to disclose more price information when blockchain costs are lower. However, as the application cost of blockchain increases, agents have no incentive to increase the disclosure of price information. While agents can increase product prices or reduce price disclosures to compensate for the cost of blockchain adoption, this approach comes at a cost, which can dampen consumer demand. That is, only reducing the cost of blockchain application is a desirable approach for agents and consumers. The reason is that the former can obtain more benefits, and the latter can obtain lower prices and more real price information.

*Lemma 2:* Comparing the amount of information disclosure and agent profit in YY, YN and NN scenarios:

1. The price information disclosure of the two agents is $\theta_{i}^{\mathrm{YY}}<\theta_{i}^{\mathrm{YN}}$; $\theta_{j}^{\mathrm{YY}}<\theta_{j}^{\mathrm{YN}}$ and$\;\theta_{\left\{i,j\right\}}^{\mathrm{YY}}>\theta_{\left\{i,j\right\}}^{\mathrm{NN}}$.
2. Since $\pi_{j}^{\mathrm{YY}}=\pi_{i}^{\mathrm{YY}}$, therefore, in the YY scenario, there is a common upper limit of the blockchain threshold ($c_{1}^{g}$).
3. There is a threshold for the cost of blockchain application ($c_{1}^{f}$), if $\;0\leq c_{B}\langle c_{1}^{f},\pi_{i}^{\mathrm{YY}}\rangle \pi_{i}^{\mathrm{YN}};$ When $c_{1}^{f}<c_{B}<c_{1}^{g},\mathrm{then},\pi_{i}^{\mathrm{YY}}<\pi_{i}^{\mathrm{YN}}$.
4. There is a threshold for the cost of blockchain application ($c_{1}^{h})$, if $0\leq c_{B}\langle c_{1}^{h},\pi_{j}^{\mathrm{YY}}\rangle \pi_{j}^{\mathrm{YN}};$ When $c_{1}^{h}<c_{B}<c_{1}^{g}$, then, $\pi_{j}^{\mathrm{YY}}<\pi_{j}^{\mathrm{YN}}$.
5. There is a threshold for the cost of blockchain application ($c_{1}^{k}$), if $\;0\leq c_{B}\langle c_{1}^{k},\pi_{\left\{i,j\right\}}^{\mathrm{YY}}\rangle \pi_{\left\{i,j\right\}}^{\mathrm{NN}}$; When $c_{1}^{k}\;$<$\;c_{B}<c_{1}^{g}$, then, $\pi_{\left\{i,j\right\}}^{\mathrm{YY}}<\pi_{\left\{i,j\right\}}^{\mathrm{NN}}$.

The expressions for $c_{1}^{f},c_{1}^{h},c_{1}^{k}\;\mathrm{and}\;c_{1}^{g}$ are shown in the Appendix.

From Lemma 2(1), it can be seen that the two agents have no incentive to release more information in the YY scenario compared to the YN scenario. Due to the cost of blockchain and the cost of information disclosure, this may obscure the benefits of applying blockchain technology, which is very disadvantageous for both agencies. However, compared to the NN scenario, the two agents reveal more information in the YY scenario. From Lemma 2(3)–(5), it can be seen that when the blockchain cost is low, both agents will gain more profit in the YY scenario than in the YN and NN scenarios. However, when the blockchain cost exceeds the threshold, the profit of the two agents in the YY scenario is less than that in the YN and NN scenarios. The reason is that the increase in demand due to the application of blockchain technology is not able to compensate the negative impact of cost increase.

## The effect of blockchain application

### Equilibrium strategy

From the above analysis, we can see that the agent’s blockchain adoption strategy is affected by the cost of blockchain application, consumers’ trust in information, and the cost of new information disclosure. By analyzing the optimal strategy after blockchain application in NN, YN, YY scenarios, we can get the following Lemma 3:

1. If $0\leq c_{B}<\left\{c_{1}^{f},c_{1}^{h},c_{1}^{k}\right\}$, the balanced strategy is that both agents apply blockchain technology (Strategy YY).
2. If $\left\{c_{1}^{a},c_{1}^{c},c_{1}^{k}\right\}<c_{B}<\left\{c_{1}^{b},c_{1,}^{d}c_{1}^{g}\right\}$, the equilibrium strategy is that neither agent applies blockchain technology (Strategy NN).
3. If $\left\{c_{1}^{f},c_{1}^{h}\right\}\leq c_{B}<\left\{c_{1}^{a},c_{1}^{c}\right\}$, for both agents, strategy YN is better than YY or NN strategy.

From Lemma 3, it can be seen that the equilibrium strategy of the two agents depends on the blockchain application cost. When the blockchain cost is very low (high), both agents choose the YY strategy or the NN strategy. When the blockchain cost is in the middle threshold, the YN strategy will be the best choice. In the YN scenario, when agent *i* applies blockchain technology, agent *j* will ignore it. Although the information on the market is full of voices, the total amount of information will be more and more accurate than in the NN scenario. The application of blockchain technology by agent *i* can solve the doubts of consumers who are more sensitive to the authenticity of information. Agent *j* who does not apply blockchain technology can meet the needs of consumers who are more sensitive to price. Therefore, the market is optimal under the supply regulation of the two agents, which also reflects the positive externality of blockchain technology. No matter which agency applies the technology, it can make both agencies better.

**Figure 4:**
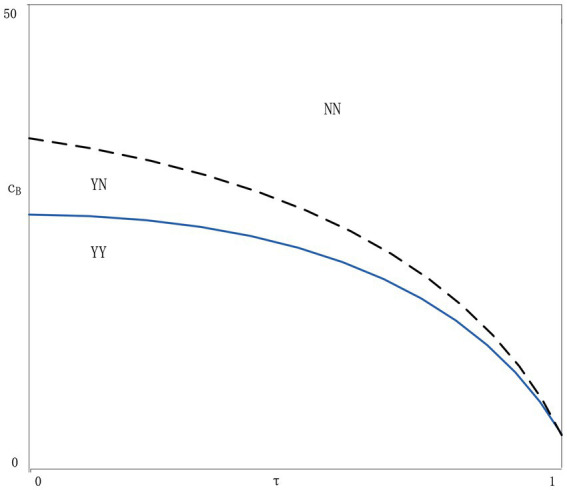
Balanced blockchain adoption strategy.

Lemma 1 to 4 only discuss the influence of consumer trust and blockchain cost on agent strategy choice. Since the specific range of blockchain costs is difficult to identify, we deploy numerical analysis to show the impact of consumer trust and blockchain costs on the equilibrium strategy. We randomly set α=100,w=5,$\;c_{p}=0.2$,β=0.5,μ=0.3, *t* = 0.2; Assuming τ and $c_{B}$ are uniformly distributed, take 20 values from 0–1, respectively. The numerical results are shown in Figure 4. The numerical simulation results are consistent with our expectations. Applying blockchain technology to improve consumer trust can make things better for at least one agent when the cost of blockchain is low. When both consumer trust and blockchain costs are high, it is best to ignore blockchain technology.

### The welfare impact of blockchain applications

**Figure 5:**
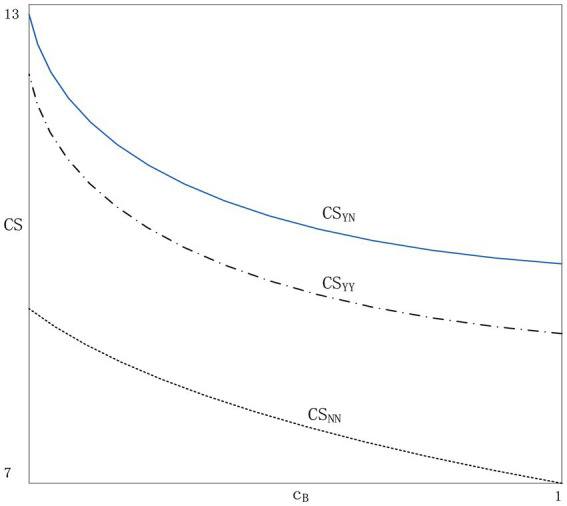
The impact on consumer welfare.

We have discussed the impact of blockchain technology on product prices, product demand and agent profits. However, the impact of blockchain technology on consumer welfare is unclear. Therefore, it is necessary to explore the impact of applying blockchain technology on consumer welfare. Due to the complexity of the model, we use numerical simulation, which can intuitively reflect the changes in consumer welfare (Lu et al., 2021). The consumer’s welfare in each of the three scenarios is obtained according to the utility function in Section “Demand function”. We set V=5, *μ* = 0.30, 0.35, 0.4, 0.45, 0.5 and 0.6; *β* = 0.4, 0.5, 0.6, 0.7, 0.8 and 0.9; We obtain consumer welfare by changing the values of consumer trust and price disclosure. Such as *τ* = 0.2, $\theta_{i}\;$ and $\theta_{j}$=0.3, take 20 discrete values from 0.2 to 1 and from 0.3 to 1, respectively. The consumer welfare under the three scenarios is shown in Figure 5.

As can be seen from Figure 5, when one agent applies the blockchain and the other agent does not, the consumer surplus is the largest (Scenario YN). Consumer surplus is the lowest (Scenario NN) when neither agent applies the blockchain. The reason is that under the YN strategy, two agents can satisfy consumers with different preferences for price and information authenticity, so the consumer surplus is the highest.

## Some extensions

### Cost-sharing contracts

Since each transaction of the agent needs to pay a certain transaction fee to the platform provider, it is beneficial for the platform to increase the number of transactions. In reality, platforms also often subsidize prices for agents to increase consumer purchases. Therefore, to encourage agents to apply blockchain technology to facilitate transaction volume, we assume that under a cost-sharing contract, both agents are willing to apply blockchain technology. Then, based on the YY scenario, we construct a cost-sharing contract (Scenario CYY). Under this contract, the application cost of the blockchain is jointly provided by the platform and the agent in proportion. The agent supports a part of the cost *η*, and the platform supports another part of the cost (1 − *η*). The profit function of the platform and the agent can be expressed as follows:


(15) $$\pi_{i}^{\mathrm{CYY}}=\left(p_{i}-w-c_{p}-\eta c_{B}\right).D_{i}^{\mathrm{YY}}-\frac{1}{2}t\theta_{i}{}^{2}\;$$

(16) $$\pi_{j}^{\mathrm{CYY}}=\left(p_{j}-w-c_{p}-\eta c_{B}\right).D_{j}^{\mathrm{YY}}-\frac{1}{2}t\theta_{j}{}^{2}$$

(17) $$\pi_{p}^{\mathrm{CYY}}=\left(c_{p}-\left(1-\eta \right)c_{B}\right).D_{T}^{\mathrm{YY}}-\frac{1}{2}t\theta_{T}{}^{2},T=i+j$$

Under the platform’s profit function, we assume that the platform transaction rate is the same for both agents. The platform transaction volume is composed of the transaction volume of the two agents together. In addition, it can be clearly known that only if the platform rate is greater than the cost sharing ratio, the platform has the motivation to implement the cost sharing contract. The platform’s profits are also affected by the rise in total transaction volume after applying the blockchain. If the increase in the total transaction volume after the application of blockchain technology is too small, that is, the total profit of the platform has not increased significantly, and the platform also lacks motivation to promote the application of blockchain technology.

In the CYY scenario, the estimated results of optimal consumer information disclosure, price, and agency profit are as follows:


$$\theta_{i}^{\mathrm{CYY}}=\theta_{j}^{\mathrm{CYY}}=\frac{\left(w+c_{p}+\eta c_{B}\right)\left(1-\beta \right)-\alpha }{1+\left(\beta -2\right)t-\mu }$$

$$p_{i}^{\mathrm{CYY}}=p_{j}^{\mathrm{CYY}}=\frac{t\alpha -\left(w+c_{p}+\eta c_{B}\right)\left(\mu -1-t\right)}{1-t\left(\beta -2\right)-\mu }$$

$$\begin{array}{c} \pi_{i}^{\mathrm{CYY}}=\pi_{j}^{\mathrm{CYY}}\\ =\frac{{\left(\alpha +\left(w+c_{p}+\eta c_{B}\right)\left(\beta -1\right)\right)}^{2}t^{3}\left(2-\beta \right)A-t\left(1-\mu \right)B}{2{\left({\left(\mu -1\right)}^{2}-t^{2}{\left(\beta -2\right)}^{2}\right)}^{2}} \end{array}$$

$$\pi_{p}^{\mathrm{CYY}}=\frac{\begin{array}{l} 2t\left(\left(w+c_{p}+\eta c_{B}\right)\left(1-\beta \right)-\alpha \right)\\ \left(\begin{array}{l} QW\left(\alpha +\left(\omega +c_{p}\right)\left(\beta -1\right)+\left(\beta -1\right)\eta c_{B}\right)\\ -JV^{2}\left(\left(\eta -1\right)c_{B}+c_{p}\right) \end{array}\right) \end{array}}{QV^{2}K}$$

**Figure 6:**
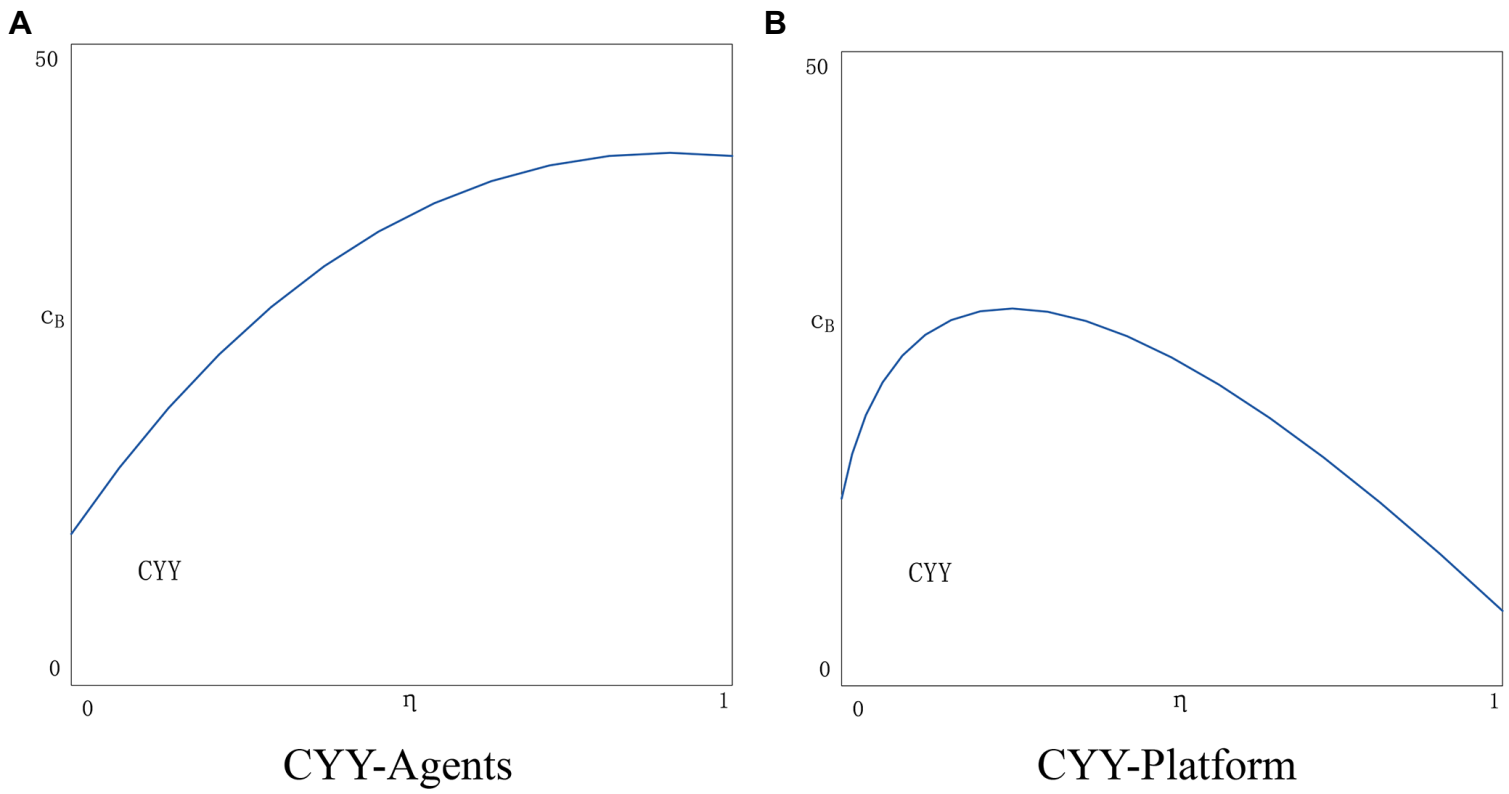
**(A)** CYY-Agents. **(B)** CYY-Platform.

Since equilibrium solutions are difficult to compute, we deploy a series of values to simulate possible solutions. Other values still refer to the settings in Section “The welfare impact of blockchain applications”. In real-world scenarios, the platform subsidizes agents usually no more than 50% of the cost. Therefore, to maintain consistency with reality, we set *η* = 0.1, 0.2, 0.3, 0.4, 0.5, respectively. The results are shown in Figure 6. As the cost-sharing ratio *η* increases, the two agents are more motivated to adopt the YY strategy (see Figure 6A). It is quite beneficial for agents to reduce the cost of blockchain application and increase consumer trust. However, an increase in *η* is quite detrimental to the platform. Figure 6B shows the situation of the platform in the CYY scenario. It can be seen that there is a threshold for the cost-sharing ratio *η*. When *η* reaches the threshold, the platform’s marginal profit decreases, and the platform may refuse to join the cost-sharing contract.

### Penalties for dishonesty

It is difficult to promote an increase in consumer purchases if the agent’s product price reduction is small. Therefore, agents have a strong incentive to adjust the initial price information to create an illusion of historically ultra-low prices in order to stimulate consumer purchases. Although the blockchain has the characteristics that it cannot be modified by one party, we assume that before applying the blockchain, the agent replaces the real price data with false prices, which means that the original price information of the blockchain system is not true. Dishonest behavior of agents is common in reality. For example, before the recent Double Eleven event, the agents adjusted the original price data of commodities in advance, resulting in the illusion of huge price reductions. The impact of agent dishonesty on blockchain application strategy will be considered. It is very possible for agents to establish false price information before the application of the blockchain system to be captured by consumers. We assume that the probability of being captured is φ(0≤φ≤1). Agents face severe penalties if dishonest practices are detected, such as cancelled orders, customer returns and regulatory fines. We denote the agent’s loss by *F*.

We use the superscripts DYN and DYY to represent the scenario where only one agent and two agents apply blockchain technology.

In the DYN scenario, the agent profit function is:


(18) $$\pi_{i}^{\mathrm{DYN}}=\left(p_{i}-w-c_{p}-c_{B}\right).D_{i}^{\mathrm{YN}}-\frac{1}{2}t\theta_{i}{}^{2}-\left(\varphi F\right)p_{i}$$

(19) $$\pi_{j}^{\mathrm{DYN}}=\left(p_{j}-w-c_{p}-c_{B}\right).D_{j}^{\mathrm{YN}}-\frac{1}{2}t\theta_{j}{}^{2}$$

In the scenario DYN, the estimated results of optimal consumer information disclosure, price, and agency profit are as follows:


$$\theta_{i}^{\mathrm{DYN}}=\frac{\left(\alpha -\left(\omega -c_{p}\right)\left(\beta +1\right)\right)B-G\left(\varphi F-c_{B}\right)}{A}$$

$$\theta_{j}^{\mathrm{DYN}}=\frac{\tau \left(\left(\alpha -\left(\omega -c_{p}\right)\left(\beta +1\right)\right)H-J\left(\varphi F-c_{B}\right)\right)}{A}$$

$$\begin{array}{c} p_{i}^{\mathrm{DYN}}=\frac{\left(\left(\omega +c_{p}\right)+\alpha \right)}{\left(2-\beta \right)}\\ +\frac{\begin{array}{l} \left(\alpha +\left(\omega +c_{p}\right)\left(\beta -1\right)\right)\\ \left(B*\beta *\mu *\tau -H\left(\beta -2\mu \right)\tau^{2}-2B\right)\\ +\left(c_{B}+\varphi *F\right)\left(2A-2G+G*\beta *\mu *\tau +J\left(\beta -2\mu \right)\tau^{2}\right) \end{array}}{A\left(4-\beta^{2}\right)} \end{array}$$

$$\begin{array}{c} p_{j}^{\mathrm{DYN}}=\frac{\left(\left(\omega +c_{p}\right)+\alpha \right)}{\left(2-\beta \right)}\\ +\frac{\begin{array}{c} \left(\alpha +\left(\omega +c_{p}\right)\left(\beta -1\right)\right)\\ \left(B\beta -2B\mu \tau +H\left(2-\beta \mu \right)\tau^{2}\right)\\ +\left(c_{B}+\varphi *F\right)\left(\begin{array}{l} -A\beta +G\beta -2G\mu \tau \\ +J\left(-2+\beta \mu \right)\tau^{2} \end{array}\right) \end{array}}{A\left(\beta^{2}-4\right)} \end{array}$$

$\pi_{i}^{\mathrm{DYN}}=\frac{\begin{array}{l} t\left(2t-1\right)\\ {\left(\begin{array}{l} \left(\alpha +\left(\omega +c_{p}\right)\left(-1+\beta \right)\right)\left(\beta^{2}-4\right)\\ \left(t\left(2+\beta \right)-\left(1+\mu \right)\tau^{2}\right)+\left(\begin{array}{l} \left(\varphi *F+c_{B}\right)\left(-4+\beta^{2}\right)\\ \left(t\left(-2+\beta^{2}\right)+\left(1-\beta \mu \right)\tau^{2}\right) \end{array}\right) \end{array}\right)}^{2} \end{array}}{2A^{2}{\left(\beta^{2}-4\right)}^{2}}$ $\pi_{j}^{\mathrm{DYN}}=\frac{\begin{array}{l} t\left(2t-\tau^{2}\right)\\ {\left(\begin{array}{l} \left(\alpha +\left(\omega +c_{p}\right)\left(-1+\beta \right)\right)\\ \left(\beta^{2}-4\right)\left(1-t\left(2+\beta \right)+\mu \tau \right)\\ +\left(\left(\varphi *F+c_{B}\right)\left(\beta^{2}-4\right)\left(\left(t-1\right)\beta +\mu \tau \right)\right) \end{array}\right)}^{2} \end{array}}{2A^{2}{\left(\beta^{2}-4\right)}^{2.}}$

In the DYY scenario, the agent profit function is:


(20) $$\pi_{i}^{\mathrm{DYY}}=\left(p_{i}-w-c_{p}-c_{B}\right).D_{i}^{\mathrm{YY}}-\frac{1}{2}t\theta_{i}{}^{2}-\left(\varphi F\right)p_{i}$$

(21) $$\pi_{j}^{\mathrm{DYY}}=\left(p_{j}-w-c_{p}-c_{B}\right).D_{j}^{\mathrm{YY}}-\frac{1}{2}t\theta_{j}{}^{2}-\left(\varphi F\right)p_{j}$$

In the DYY scenario, the estimated results of optimal consumer information disclosure, price, and agency profit are as follows:


$$\theta_{i}^{\mathrm{DYY}}=\theta_{j}^{\mathrm{DYY}}=\frac{\alpha +\left(\omega +c_{p}+c_{B}+\varphi F\right)\left(\beta -1\right)}{1-2t+t\beta -\mu }$$

$$\begin{array}{c} p_{i}^{\mathrm{DYY}}=p_{j}^{\mathrm{DYY}}\\ =\frac{t\left(\left(\omega +c_{p}+c_{B}+\varphi F\right)+\alpha \right)}{t\left(2-\beta \right)+\mu -1}\\ \;\;+\frac{\left(2\alpha +\left(\omega +c_{p}+c_{B}+\varphi F\right)\beta \right)\left(\mu -1\right)}{\left(\beta -2\right)\left(1+t\left(-2+\beta \right)-\mu \right)} \end{array}$$

$$\begin{array}{c} \pi_{i}^{\mathrm{DYY}}=\pi_{j}^{\mathrm{DYY}}\\ =\frac{\begin{array}{l} {\left(\alpha +\left(\omega +c_{p}+c_{B}+\varphi F\right)\left(\beta -1\right)\right)}^{2}\\ \left(t\left(2-\beta \right)\left(2t\left(2-\beta \right)-10+\beta +8\mu \right)+8{\left(1-\mu \right)}^{2}\right) \end{array}}{2{\left(\beta -2\right)}^{2}{\left(1+t\left(\beta -2\right)-\mu \right)}^{2}} \end{array}$$

Again, we choose numerical simulation to obtain the equilibrium solution. The probability that an agent’s dishonest behavior is detected is maximized to 1 and minimized to 0. We set φ∈{0~1}. According to the Chinese Consumer Protection Law, the maximum penalty for dishonest behavior is 3 times the price of the product, so we set *F* = 3.

**Figure 7:**
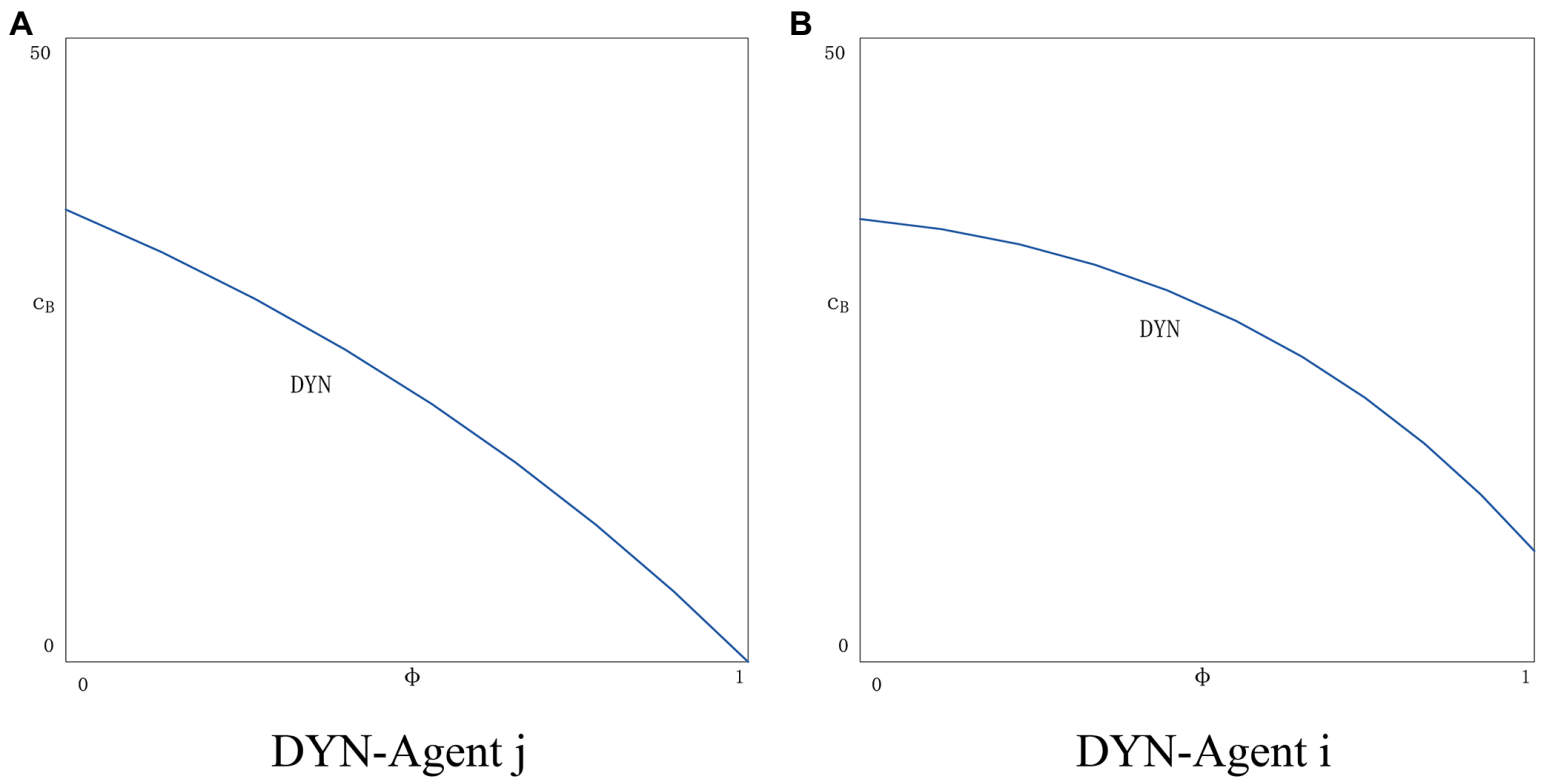
**(A)** DYN-Agent *j*. **(B)** DYN-Agent *i*.

**Figure 8:**
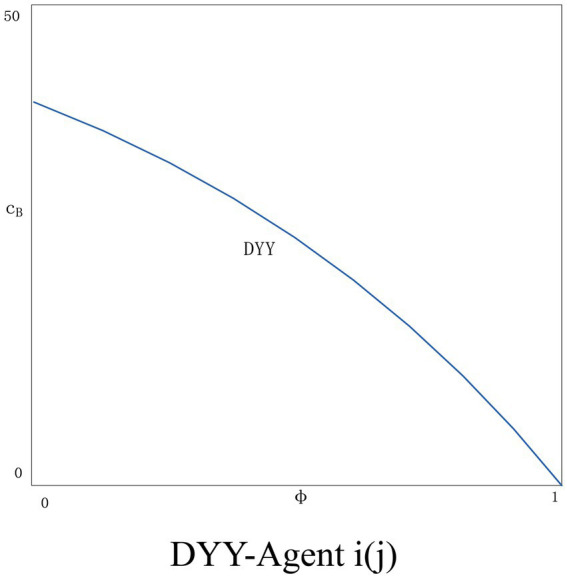
DYY-Agent *i*(*j*).

In the DYN scenario, there is no significant correlation between policy choice and the probability of dishonest behavior being detected as agent *j* does not apply blockchain technology. However, as the probability of dishonest behavior being detected increases, agent *j* will be implicated. As shown in Figure 7A, the situation of agent *j* will get worse as φ increases. The situation for agent *i* will get better (see Figure 7B). As the value of φ becomes larger, agent *i* pays a heavy price for dishonest behavior (i.e., high fines), which constrains agent *i*’s behavior to provide completely truthful information. The information of agent *j* is still unauthenticated. In the DYY scenario (see Figure 8), the two agents have exactly the same chance of being penalized for dishonesty. As *φ* rises, there is a huge risk of dishonesty, and both agents are more cautious. In general, with the introduction of the punishment policy for dishonest behavior on the platform, the price information in the market is true and reliable, and the consumer trust and transaction volume both increase together, and finally achieve a virtuous circle, which is conducive to the development of the platform.

### Variable blockchain costs

In the previous analysis, we assumed that the unit application cost of the blockchain is fixed. However, in reality, the blockchain cost is divided into two parts (fixed cost and variable cost). The fixed cost is the initial system construction cost of each product. Variable costs increase with the amount of information disclosed. We denote fixed costs by $c_{B}$ and variable costs by $c_{vB}$. We use the superscripts VYN and VYY to denote two scenarios of variable blockchain costs.

When only one agent applies blockchain technology, the profit function can be expressed as follow:


(22) $$\pi_{i}^{\mathrm{VYN}}=\left(p_{i}-w-c_{p}-c_{B}-\left(c_{vB}\theta_{i}\right)\right).D_{i}^{\mathrm{VYN}}-\frac{1}{2}t\theta_{i}{}^{2}$$

(23) $$\pi_{j}^{\mathrm{VYN}}=\left(p_{j}-w-c_{p}\right).D_{j}^{\mathrm{VYN}}-\frac{1}{2}t\theta_{j}{}^{2}$$

In the VYN scenario, the estimated results of optimal consumer information disclosure, price, and agency profit are as follows:


$$\theta_{i}^{\mathrm{VYN}}=\frac{\left(\alpha +\left(\omega +c_{p}\right)\left(\beta -1\right)\right)B+F\left(c_{B}+c_{vB}\right)}{A}$$

$$\theta_{j}^{\mathrm{VYN}}=\frac{\left(\alpha +\left(\omega +c_{p}\right)\left(\beta -1\right)\right)G+H\left(c_{B}+c_{vB}\right)}{A}$$

$$\begin{array}{c} p_{i}^{\mathrm{VYN}}=\frac{\left(\left(\omega +c_{p}\right)+\alpha \right)}{\left(2-\beta \right)}\\ +\frac{\begin{array}{l} \left(\alpha +\left(\omega +c_{p}\right)\left(\beta -1\right)\right)\left(B\left(\beta \mu \tau -2\right)+G\tau \left(2\mu -\beta \right)\right)\\ +\left(\mu \tau \left(2H+F\beta \right)-2\left(A+F\right)-H\beta \tau \right)\left(c_{B}+c_{\mathrm{vB}}\right) \end{array}}{A\left(\beta^{2}-4\right)} \end{array}$$

$$\begin{array}{c} p_{j}^{\mathrm{VYN}}=\frac{\left(\left(\omega +c_{p}\right)+\alpha \right)}{\left(2-\beta \right)}\\ +\frac{\begin{array}{l} \left(\alpha +\left(\omega +c_{p}\right)\left(\beta -1\right)\right)\\ \left(G\left(\beta \mu -2\right)\tau -B\left(\beta -2\mu \tau \right)\right)\\ -\left(\left(A+F\right)\beta +2H\tau -\left(2F+H\beta \right)\mu \tau \right)\left(c_{B}+c_{\mathrm{vB}}\right) \end{array}}{A\left(\beta^{2}-4\right)} \end{array}$$

When both agents apply blockchain technology, the profit function can be expressed as:


(24) $$\pi_{i}^{\mathrm{VYY}}=\left(p_{i}-w-c_{p}-c_{B}-\left(c_{vB}\theta_{i}\right)\right).D_{i}^{\mathrm{VYN}}-\frac{1}{2}t\theta_{i}{}^{2}$$

(25) $$\pi_{j}^{\mathrm{VYY}}=\left(p_{i}-w-c_{p}-c_{B}-\left(c_{vB}\theta_{j}\right)\right).D_{j}^{\mathrm{VYN}}-\frac{1}{2}t\theta_{j}{}^{2}$$

In the scenario VYY, the estimated results of optimal consumer information disclosure, price, and agency profit are as follows:


$$\theta_{i}^{\mathrm{VYY}}=\theta_{j}^{\mathrm{VYY}}=\frac{\left(\omega +c_{p}+c_{B}+c_{vB}\right)\left(1-\beta \right)-\alpha }{1+\left(\beta -2\right)t-\mu }$$

$$p_{i}^{\mathrm{VYY}}=p_{j}^{\mathrm{VYY}}=\frac{t\alpha -\left(\omega +c_{p}+c_{B}+c_{vB}\right)\left(\mu -1-t\right)}{1-t\left(\beta -2\right)-\mu }$$

$$\begin{array}{c} \pi_{i}^{\mathrm{VYY}}=\pi_{j}^{\mathrm{VYY}}\\ =\frac{\begin{array}{l} {\left(\alpha +\left(\omega +c_{p}+c_{B}+c_{vB}\right)\left(\beta -1\right)\right)}^{2}\\ t^{3}\left(2-\beta \right)A-t\left(1-\mu \right)B \end{array}}{2{\left({\left(\mu -1\right)}^{2}-t^{2}{\left(\beta -2\right)}^{2}\right)}^{2}} \end{array}$$

Since there are too many unknown parameters, a balanced strategy is difficult to obtain. Refer to previous research, we introduce numerical simulations to obtain approximate possible solutions. The variable cost per unit is lower than the fixed cost, we assign the variable cost $c_{vB}$ as 0.02, 0.05, 0.07, 0.09, 0.1, and 0.12.

**Figure 9:**
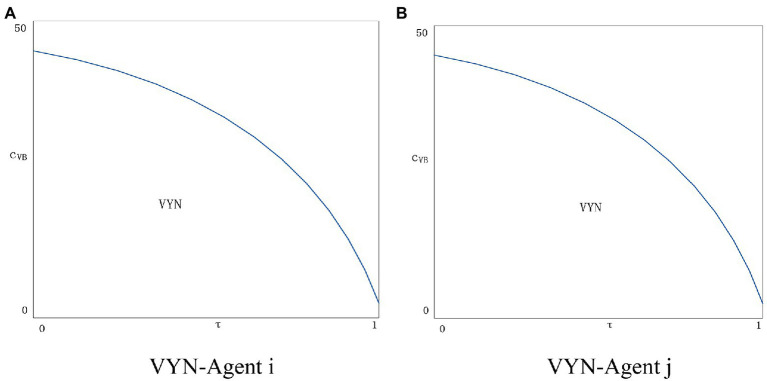
**(A)** VYN-Agent *i*. **(B)** VYN-Agent *j*.

**Figure 10:**
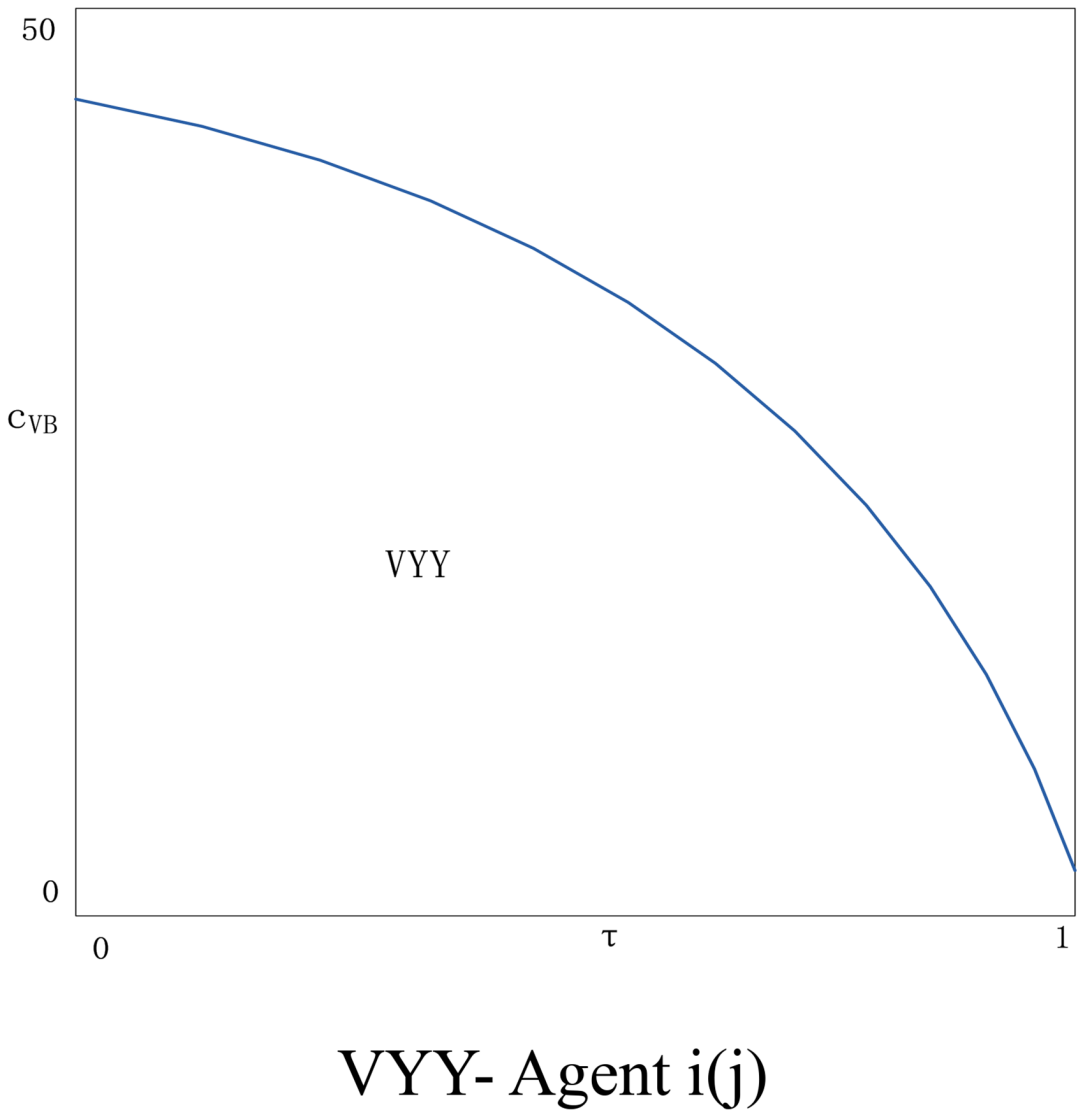
VYY- Agent *i*(*j*).

As can be seen from Figure 9, after adding the variable cost of the blockchain, the situation of the two agents becomes better when the variable cost is lower. The agent *i* applying the blockchain technology increases the profit as the consumer’s trust in the price information increases. Agents *j* who do not apply blockchain technology will increase the amount of information disclosure to resist the threat from the advantages of blockchain application. As the blockchain variable cost exceeds a certain threshold, the situation of both agents will get worse, and the situation of agent *i* will be even worse. With the increase in the amount of information disclosure, the variable cost of blockchain will continue to rise, and the agent *i* will be overwhelmed and will not choose to apply blockchain technology. At the same time, the attractiveness of agent *j* to disclose new information to consumers will gradually decline, and none of them will be able to benefit in this situation. Figure 10 shows the situation of two agents applying blockchain technology, it can be found that as the variable cost rises, the situation of both agents becomes worse, therefore, no agent has the incentive to apply the blockchain technology.

## Conclusion and management implications

### Conclusion

With the increasing number of agents on the platform, the price competition among agents has become more intense. Agents often use low price strategies to attract consumers. However, the low price strategy is often filled with false information and consumers perceive the non-truthfulness of the price information. Then, consumers’ trust in agents gradually decreases, which inhibits the growth of online shopping. Blockchain is seen as a solution to the trust crisis between agents and consumers. Our research is based on two competing agents selling the same type of goods on the same platform. Agents can freely set commodity price information and publish various price information and advertisements in their own stores on the platform. We discussed the blockchain technology application strategies of agents in three scenarios: (1) neither agent introduced blockchain technology (Strategy NN); (2) only agent *i* introduced blockchain technology mode (Strategy YN); (3) Both agents introduce blockchain technology (Strategy YY). By comparing the price information disclosure and the agent’s profit under the three strategies. We got the agent’s blockchain technology application strategy, and identified important influencing factors. The results show that the application of blockchain technology is beneficial to agents only when consumer trust is low. Furthermore, the YN strategy is regarded as a possible equilibrium strategy, which depends on the blockchain application cost and consumer trust. Some extended cases are discussed for post-blockchain consumer welfare, cost-sharing contracts, dishonesty penalties, and variable blockchain costs, and the results show that the analysis in this manuscript is robust. Our findings have important practical significance for promoting the application of blockchain technology and alleviating the problem of price information asymmetry in platform shopping.

### Management implications

The manuscript assumes that blockchain technology affects information disclosure costs and consumer trust, and that consumers can switch freely between the two agents. However, the reality is that agents use a variety of methods to create stickiness with consumers, such as membership and progressive discounts. Consumers need to pay a certain cost when switching purchase channels. Future research can be further extended to the impact of purchase channels and consumer multi-attribution. The application of blockchain in agent price management is still in the exploratory stage. Based on the findings of this manuscript, the following recommendations are presented:First, the fixed cost of blockchain technology application is scientifically controlled to realize the improvement of consumer trust and agent profit, so as to give full play to the positive externality of blockchain. Second, the application specifications of blockchain technology should be improved to ensure that consumers’ trust can be truly enhanced and avoid some agents from using loopholes to commit dishonest behavior. Finally, platforms can take the lead in developing blockchain pilot areas to promote the enthusiasm of agents to apply blockchain technology. By piloting regional chain technology in certain areas where consumer trust is extremely low, it can better serve consumers and agents and promote the growth of platform shopping transactions.

## Data availability statement

Publicly available datasets were analyzed in this study. This data can be found at: https://data.worldbank.org/.

## Author contributions

LW: manuscript revision, software, methodology, and project administration. GX: writing–original draft, formal analysis, funding acquisition, and supervision. CC: manuscript revision, literature search, formal analysis, visualization, and funding acquisition. All authors contributed to the article and approved the submitted version.

## Funding

This work was supported by the Major Program of National Social Science Foundation of China (21&ZD121).

## Conflict of interest

The authors declare that the research was conducted in the absence of any commercial or financial relationships that could be construed as a potential conflict of interest.

## Publisher’s note

All claims expressed in this article are solely those of the authors and do not necessarily represent those of their affiliated organizations, or those of the publisher, the editors and the reviewers. Any product that may be evaluated in this article, or claim that may be made by its manufacturer, is not guaranteed or endorsed by the publisher.
