# Supplementary material for: Price competition and blockchain technology adoption strategies of agents on the digital platform
Source: Front Psychol. 2022 Oct 14;13:984928. doi: 10.3389/fpsyg.2022.984928 (PMC9615551; doi:10.3389/fpsyg.2022.984928)
Supplement: Supplementary file 1 [file Data_Sheet_1.pdf]

Appendix

Under the strategy NN:

The revenue functions of agents i and j are:

$$\pi_i^{NN} = (p_i - w - c_p).(\alpha - p_i + \beta p_j + \tau(\theta_i - \mu\theta_j)) \;\; -\frac{1}{2}t\theta_i{}^2$$

$$\pi_j^{NN} = (p_j - w - c_p).\alpha - p_j + \beta p_i + \tau(\theta_j - \mu\theta_i) \;\; -\frac{1}{2}t\theta_j{}^2$$

The corresponding first partial derivatives are:

$$\frac{\partial \pi_i^{NN}}{\partial p_i} = w + \alpha + c_p - 2p_i + \beta p_j + \tau(\theta_i - \mu\theta_j)$$

$$\frac{\partial \pi_j^{NN}}{\partial \theta_j} = w + \alpha + c_p - 2p_j + \beta p_i + \tau(\theta_j - \mu\theta_i)$$

$$\frac{\partial \pi_i^{NN}}{\partial \theta_i} = \tau(p_i - w - c_p) - t\theta_i$$

$$\frac{\partial \pi_j^{NN}}{\partial \theta_j} = \tau(p_j - w - c_p) - t\theta_j$$

Letting $\frac{\partial \pi_i^{NN}}{\partial p_i}$=0， $\frac{\partial \pi_j^{NN}}{\partial p_j} = 0$， $\frac{\partial \pi_i^{NN}}{\partial \theta_i}$=0， $\frac{\partial \pi_j^{NN}}{\partial \theta_j}$=0, the optimal sales price and the corresponding optimal price information disclosure quantity are obtained as follows.

$$p_i^{NN}, p_j^{NN} = \frac{t\alpha + (t + (\mu - 1)\tau^2)(w + c_p)}{t(2-\beta) + (\mu - 1)\tau^2}, \theta_i^{NN} = \theta_j^{NN} = \frac{\tau(\alpha + (\beta - 1)(w + c_p))}{t(2-\beta) + (\mu - 1)\tau^2}$$

Substituting the optimal sales price and optimal price information into the target's revenue function, we can get the maximum profit of the two agents:

$$\pi_i^{NN} = \pi_j^{NN} = \frac{t(2t - \tau^2)(\alpha + (\beta - 1)(w + c_p))^2}{2(t(2-\beta)) + (\mu - 1)\tau^2)^2}$$

Proof of Corollary 1.

$\frac{dD_i^{NN}}{\partial \tau} = \frac{dD_j^{NN}}{\partial \tau} = \frac{2t(\mu+1)\tau(\alpha - (\beta+1)(\omega - c_p))}{(t(\beta-2) - (\mu-1)\tau^2)^2}$, we know $\alpha \geq 1$, we have $(\alpha - (\beta + 1)(\omega - c_p)) > 0$, then $\frac{dD_{\{i,j\}}}{d\tau} > 0$;

$\frac{dp_i^{NN}}{d\tau} = \frac{dp_j^{NN}}{d\tau} = \frac{2t(\mu+1)\tau(\alpha - (\beta+1)(\omega - c_p))}{(t(\beta-2) - (\mu-1)\tau^2)^2}$, we know $\alpha \geq 1$, and $(\alpha - (\beta + 1)(\omega - c_p)) > 0$, then $\frac{dp_{\{i,j\}}^{NN}}{d\tau} > 0$;

$\frac{d\theta_i^{NN}}{d\tau} = \frac{d\theta_j^{NN}}{d\tau} = \frac{(t(\beta+2) + (\mu+1)\tau^2)(\alpha - (\beta+1)(\omega - c_p))}{(t(\beta-2) - (\mu-1)\tau^2)^2}$, we know $\alpha \geq 1$, and $(\alpha - (\beta +$

$1)\left(\omega - c_p\right)\big) > 0$, then $\frac{d\theta_{i,j}^{NN}}{d\tau} > 0$;

$\frac{d\pi_i^{NN}}{d\tau} = \frac{d\pi_j^{NN}}{d\tau} = \frac{t\tau(t(2-\beta+4\mu)-(\mu+1)\tau^2)(\alpha-(\beta+1)\omega-(\beta+1)c_p)^2}{(t(\beta-2)-(\mu-1)\tau^2)^3}$,we know, $(\alpha-(\beta+1)\omega-(\beta+1)c_p)^2 > 0$, so, we set $\tau^* = t\tau(t(2-\beta+4\mu)$ and $\tau = (\mu+1)\tau^2$,when $\tau < \tau^*$,$\frac{d\pi_{\{i,j\}}^{NN}}{d\tau} > 0$, otherwise, $\frac{d\pi_{\{i,j\}}^{NN}}{d\tau} < 0$.

Note: In line with reality, we assume that consumers are more sensitive to cross price than to cross information disclosure, and that disclosure is profitable.

Under the NN strategy:

The revenue functions of agents i and j are:

$$\pi_i^{YN} = \left(p_i - w - c_p - c_B\right).D_i^{YN} - \frac{1}{2}t{\theta_i}^2$$

$$\pi_j^{YN} = \left(p_j - w - c_p\right).D_j^{YN} - \frac{1}{2}t{\theta_j}^2$$

The corresponding first partial derivatives are:

$$\frac{\partial\pi_i^{YN}}{\partial p_i} = w + \alpha + c_p - 2p_i + \beta p_j + \tau\left(\theta_i - \mu\theta_j\right)$$

$$\frac{\partial\pi_j^{YN}}{\partial p_j} = w + \alpha + c_p - 2p_j + \beta p_i + \tau\left(\theta_j - \mu\theta_i\right)$$

$$\frac{\partial\pi_i^{YN}}{\partial\theta_i} = \tau\left(p_i - w - c_p\right) - t\theta_i$$

$$\frac{\partial\pi_j^{YN}}{\partial\theta_j} = \tau\left(p_j - w - c_p\right) - t\theta_j$$

In order to facilitate the calculation, we first need to set some useful formulas, as follows.

$A = t(2(1+\tau^2) - t(4-\beta^2) - \beta\mu\tau(1+\tau)) + \tau^2(\mu^2\tau - 1)$

$B = (1+\mu)\tau^2 - t(2+\beta)$

$F = t(2-\beta^2) + (\beta * \mu - 1)\tau^2$

$G = \tau(1 - t(2+\beta) + \mu * \tau)$

$H = \tau(\beta(1-t) - \mu * \tau)$

U1=$(\alpha + (w + c_p)(\beta - 1))(B(\beta\mu\tau - 2) + G\tau(2\mu - \beta)) + (\mu\tau(2H + FB) - 2(A + F) - HB\tau)c_B$

U2=$(\alpha + (w + c_p)(\beta - 1)) + G\tau(\beta\mu - 2)) - B(\beta - 2\mu\tau) - ((A + F)\beta + 2H\tau - (2F + H\beta)\mu\tau)c_B$

$$U3 = t(2t-1)((\alpha + (\omega + c_p)(\beta - 1))(\beta^2 - 4)(t(2+\beta) - (1+\mu)\tau^2) + c_B(\beta^2 - 4)(t(\beta^2 - 2) + (1 - \beta\mu)\tau^2))^2;$$

$$U4 = t(2t - \tau^2)((\alpha + (\omega + c_p)(\beta - 1))(\beta^2 - 4)(1 - t(2+\beta) + \mu\tau) + c_B(\beta^2 - 4)((t-1)\beta + \mu\tau))^2;$$

Letting $\frac{\partial \pi_i^{YN}}{\partial p_i}$=0, $\frac{\partial \pi_j^{YN}}{\partial p_j} = 0$, $\frac{\partial \pi_i^{YN}}{\partial \theta_i}$=0, $\frac{\partial \pi_j^{YN}}{\partial \theta_j}$=0, the optimal sales price and the corresponding optimal price information disclosure quantity are obtained as follows.

$$p_i^{YN} = \frac{w + c_p + \alpha}{(2-\beta)} + \frac{U1}{A(\beta^2-4)} \text{ ; } p_j^{YN} = \frac{w + c_p + \alpha}{(2-\beta)} + \frac{U2}{A(\beta^2-4)}$$

$$\theta_i^{YN} = \frac{(\alpha+(w+c_p)(\beta-1))B+Fc_B}{A} \text{ ; } \theta_j^{YN} = \frac{(\alpha+(w+c_p)(\beta-1))G+Hc_B}{A}$$

Substituting the optimal sales price and optimal price information into the target's revenue function, we can get the maximum profit of the two agents:

$$\pi_i^{YN} = \frac{t(2t-1)((\alpha+(w+c_p)(\beta-1))(\beta^2-4)(t(2+\beta)-(1+\mu)\tau^2)+c_B(\beta^2-4)(t(\beta^2-2)+(1-\beta\mu)\tau^2))^2}{2A^2(\beta^2-4)^2}$$

$$\pi_j^{YN} = \frac{t(2t-\tau^2)((\alpha+(w+c_p)(\beta-1))(\beta^2-4)(1-t(2+\beta)+\mu\tau)+c_B(\beta^2-4)((t-1)\beta+\mu\tau))^2}{2A^2(\beta^2-4)^2}$$

Proof of Corollary 2.

Part I, the impact of consumer trust:

$\frac{d\theta_i^{YN}}{d\tau} = \frac{(\alpha+c_p w(\beta-1))(2\tau(\beta\mu-1)c_B+(1+\mu))}{\tau(3\mu^2\tau-2+4t(2-t(4+\beta^2)+\beta\mu\tau+2\tau^2))}$; We know$\beta \le 1$,then, $\beta - 1 \le 0$, since $\alpha \ge 1$,thus, the first term is positive as $(\alpha + c_p w(\beta-1)) > 0$. When $\beta\mu \ge 1, \frac{d\theta_i^{YN}}{d\tau} > 0$; Oterwise,$\frac{d\theta_i^{YN}}{d\tau} < 0$.

$\frac{d\theta_j^{YN}}{d\tau} = \frac{(\alpha+(w+c_p)(\beta-1))(\tau(1-2t+\beta)c_B}{\tau(3\mu^2\tau-2+4t(2-t(4+\beta^2)+\beta\mu\tau+2\tau^2))}$; We know $\beta \le 1$,then, $\beta - 1 \le 0$, since $\alpha \ge 1$, thus, the first term is positive as $(\alpha + (w+c_p)(\beta-1)) > 0$. When $t < \beta, \frac{d\theta_j^{YN}}{d\tau} > 0$; Otherwie,$\frac{d\theta_j^{YN}}{d\tau} < 0$;

$\frac{dp_i^{YN}}{d\tau} = \frac{w(2-\beta\mu\tau)c_p+\tau(2\mu-\mu\beta))+((2\beta\tau+\mu)+(1-\beta))c_B(\alpha(1-\mu)^2t(\beta-2\mu))}{\tau(3\mu^2\tau-2+4t(2-t(4+\beta^2)+\beta\mu\tau+2\tau^2))}$; We have $\beta \le 1$, $\mu \le 1, and\ \tau \le 1$, thus $2 - \beta\mu\tau > 0$, $2\mu - u\beta > 0, 1-\beta \ge 0$, and $1-\mu > 0$; thus, the range of the solution depends on $\beta - 2\mu$, when $\beta \ge 2\mu$, $\frac{dp_i^{YN}}{d\tau} > 0$; Otherwise, $\frac{dp_i^{YN}}{d\tau} < 0$.

$\frac{dp_j^{YN}}{d\tau} = \frac{w(\beta-2\mu)\tau(2-\mu)c_B(1-\beta)+(\alpha+w(\beta-1)^2c_p t(2-\beta\tau)c_p}{\tau(3\mu^2\tau-2+4t(2-t(4+\beta^2)+\beta\mu\tau+2\tau^2))}$; We have $\beta \le 1$, $\mu \le 1$, $and\ \tau \le 1$, thus $2-\mu > 0, 1-\beta \ge 0, (\beta-1)^2 > 0, and\ 2-\beta\tau > 0; then,$ the range of the solution depends on $\beta - 2\mu$, when $\beta \ge 2\mu$, $\frac{dp_j^{YN}}{d\tau} > 0$; Otherwise, $\frac{dp_j^{YN}}{d\tau} < 0$.

$\frac{d\pi_i^{YN}}{d\tau} = \frac{\alpha(4-\beta^2)(1-\mu+t(2+\beta))(4-\beta^2)\omega(1-\mu+t(2+\beta))+(4-\beta^2)c_p(1-\mu+t(2-\beta))(\beta-2\mu)}{\tau(3\mu^2\tau-2+4t(2-t(4+\beta^2)+\beta\mu\tau+2\tau^2))}$;We have $\beta \leq 1$ and $\mu \leq 1$, thus $(4-\beta^2) > 0, 1-\mu > 0$ and $2-\beta > 0$; then, the range of the solution depends on $\beta \geq 2\mu$, when $\beta \geq 2\mu$, $\frac{d\pi_i^{YN}}{d\tau} > 0$; otherwise, $\frac{d\pi_i^{YN}}{d\tau} < 0$.

$\frac{d\pi_j^{YN}}{d\tau} = \frac{2(\beta^2-4)^2+(t(2\tau^2-(4-\beta^2)t-\beta\mu\tau(1+\tau)+\tau^2(\mu^2\tau-1))^2)}{\tau(3\mu^2\tau-2+4t(2-t(4+\beta^2)+\beta\mu\tau+2\tau^2))}$; We have $(\beta^2-4)^2 > 0$, Similarly, $(t(2\tau^2-(4-\beta^2)t-\beta\mu\tau(1+\tau)+\tau^2(\mu^2\tau-1))^2) > 0$, then $\frac{\partial\pi_j^{YN}}{\partial\tau} > 0$.

Part II, the impact of blockchain application costs:

$\frac{d\theta_i^{YN}}{dc_B} = \frac{t(2-\beta^2)+(\beta\mu-1)\tau^2}{t(2(1+\tau^2)-t(4-\beta^2)-\beta\mu\tau(1+\tau))+\tau^2(\mu^2\tau-1)}$; We know $\beta \leq 1$, thus, $2-\beta^2 > 0$, then, the range of the solution depends on $\beta\mu - 1$, when $\beta\mu \geq 1$, $\frac{d\theta_i^{YN}}{dc_B} > 0$; Otherwise $\frac{d\theta_i^{YN}}{dc_B} < 0$.

$\frac{d\theta_j^{YN}}{dc_B} = \frac{\tau(\beta+\beta t-\mu t)}{t(2(1+\tau^2)-t(4-\beta^2)-\beta\mu\tau(1+\tau))+\tau^2(\mu^2\tau-1)}$; We know $\beta \leq 1$ and $\mu \leq 1$, the range of the solution depends on $\beta + \beta t - \mu t$, when $\beta > \mu$, $\frac{d\theta_j^{YN}}{dc_B} > 0$; Otherwise, $\frac{d\theta_j^{YN}}{dc_B} < 0$.

$\frac{dp_i^{YN}}{dc_B} = \frac{t\tau^2+2t^2(\beta\mu\tau-t\beta\mu\tau)+\tau^2-\mu^2\tau^3}{t(2(1+\tau^2)-t(4-\beta^2)-\beta\mu\tau(1+\tau))+\tau^2(\mu^2\tau-1)}$; We know $t < 1$, thus, we have $\beta\mu\tau > t\beta\mu\tau$. Similarly, as $\mu < 1$, we have $\tau^2 > \mu^2\tau^3$, then $\frac{dp_i^{YN}}{dc_B} > 0$.

$\frac{dp_j^{YN}}{dc_B} = \frac{t^2+2u(\beta\tau-\mu\tau)+\tau-\mu\tau}{t(2(1+\tau^2)-t(4-\beta^2)-\beta\mu\tau(1+\tau))+\tau^2(\mu^2\tau-1)}$; We know $\mu < 1$ and $\tau < 1$, thus we have $\tau > \mu\tau$, the range of the solution depends on $\beta\tau - \mu\tau$, when $\beta > \mu$, $\frac{dp_j^{YN}}{dc_B} > 0$; otherwise, $\frac{dp_j^{YN}}{dc_B} < 0$.

$\frac{dD_i^{YN}}{dc_B} = \frac{t((\beta\mu-1)\tau^2+t(2-\beta^2))}{t(2(1+\tau^2)-t(4-\beta^2)-\beta\mu\tau(1+\tau))+\tau^2(\mu^2\tau-1)}$; We know $\beta \leq 1$, thus, $2-\beta^2 > 0$, the range of the solution depends on $\beta\mu - 1$, when $\beta\mu \geq 1$, $\frac{dD_i^{YN}}{dc_B} > 0$; Otherwise, $\frac{dD_i^{YN}}{dc_B} < 0$.

$\frac{dD_j^{YN}}{dc_B} = \frac{t(\beta\tau-\mu\tau+t\beta)}{t(2(1+\tau^2)-t(4-\beta^2)-\beta\mu\tau(1+\tau))+\tau^2(\mu^2\tau-1)}$; We know $\mu < 1$ *and* $t < 1$, thus, the

range of the solution depends on $\beta\tau - \mu\tau$, when $\beta > \mu$, $\frac{dD_j^{YN}}{dc_B} > 0$; otherwise, $\frac{dD_j^{YN}}{dc_B} < 0$.

$\frac{d\pi_i^{YN}}{dc_B} = \frac{t(3-2t)+(\beta-1)+c_B\left[(\beta\mu-1)\tau^2-t(\beta^2-2)\right]^2[(\alpha+(\beta-1)(\omega+c_p)((1+\mu)\tau^2]}{(t(2(1+\tau^2)-t(4-\beta^2)-\beta\mu\tau(1+\tau))+\tau^2(\mu^2\tau-1))^2}$; We know $t < 1$, thus,
$3 - 2t > 0$. Furthermore, we have $[(\beta\mu - 1)\tau^2 - t(\beta^2 - 2)]^2 > 0$, the range of the solution depends on $\beta - 1$, when $\beta > 1$, $\frac{d\pi_i^{YN}}{dc_B} > 0$; otherwise, $\frac{d\pi_i^{YN}}{dc_B} < 0$.

$\frac{d\pi_j^{YN}}{dc_B} = \frac{2t(t+1)t\beta(2t-\tau^2)c_B}{(t(2(1+\tau^2)-t(4-\beta^2)-\beta\mu\tau(1+\tau))+\tau^2(\mu^2\tau-1))^2}$; We know $t < 1$, the range of the solution depends on $2t - \tau^2$, when $2t > \tau$, then, $\frac{d\pi_j^{YN}}{dc_B}$>0; Otherwise, $\frac{d\pi_j^{YN}}{dc_B}$<0.

Under the strategy YY:

The revenue functions of agents i and j are:

$$\pi_i^{YY} = (p_i - w - c_p - c_B)(\alpha - p_i + \beta p_j + \theta_i - \mu\theta_j) - \frac{1}{2}t{\theta_i}^2$$

$$\pi_j^{YY} = (p_j - w - c_p - c_B)(\alpha - p_j + \beta p_i + \theta_j - \mu\theta_i) - \frac{1}{2}t{\theta_j}^2$$

The corresponding first partial derivatives are:

$$\frac{\partial\pi_i^{YY}}{\partial p_i} = w + \alpha + c_B + c_p - 2p_i + \beta p_j + \theta_i - \mu\theta_j$$

$$\frac{\partial\pi_j^{YY}}{\partial p_j} = w + \alpha + c_B + c_p - 2p_j + p\beta_i + \theta_j - \mu\theta_i$$

$$\frac{\partial\pi_i^{YY}}{\partial\theta_i} = (p_i - w - c_B - c_p) - t\theta_i$$

$$\frac{\partial\pi_j^{YY}}{\partial\theta_j} = (p_j - w - c_B - c_p) - t\theta_j$$

Letting $\frac{\partial\pi_i^{YY}}{\partial p_i}$=0, $\frac{\partial\pi_i^{YY}}{\partial\theta_i} = 0$, $\frac{\partial\pi_j^{YY}}{\partial p_j}$=0, $\frac{\partial\pi_j^{YY}}{\partial\theta_j}$=0, the optimal sales price and the corresponding optimal price information disclosure quantity are obtained as follows.

$$p_i^{YY} = p_j^{YY} = \frac{t\alpha-(w+c_p+c_B)(\mu-1-t)}{1+(2-\beta)t-\mu}$$

$$\theta_i{}^{YY} = \theta_j{}^{YY} = \frac{(w+c_p+c_B)(1-\beta)-\alpha}{1+(2-\beta)t-\mu}$$

Substituting the optimal sales price and optimal price information into the target's revenue function, we can get the maximum profit of the two agents:

$$\pi_i^{YY} = \pi_j^{YY} = \frac{(\alpha + (w + c_p + c_B)(\beta - 1))^2 t^3 (2-\beta)A - t(1-\mu)B}{2((\mu-1)^2 - t^2(\beta-2)^2)^2}$$

Proof of Corollary 3.

$\frac{d\theta_i^{YY}}{dc_B} = \frac{d\theta_j^{YY}}{dc_B} = \frac{1-\beta}{1+t(2-\beta)-\mu}$; the range of the solution depends on β, When $\beta < 1$, then, $\frac{d\theta_{\{i,j\}}^{YY}}{dc_B} > 0$; Otherwise, $\frac{d\theta_{\{i,j\}}^{YY}}{dc_B} < 0$.

$\frac{dp_i^{YY}}{dc_B} = \frac{dp_j^{YY}}{dc_B} = \frac{1+t-\mu}{1+t(2-\beta)-\mu}$; We know $\mu < 1$, we have $1 + t - \mu > 0$, then, $\frac{dp_{i,j}^{YY}}{dc_B} > 0$.

$\frac{dD_i^{YY}}{dc_B} = \frac{dD_j^{YY}}{dc_B} = \frac{t(\beta+1)(t(\beta-2)-(3-2\beta)(1-\mu))}{(1-t(\beta-2)-\mu)(1+t(\beta-2)-\mu)}$; We know $\beta < 1$, thus, $\beta - 2 < 0$; We have $(3-2\beta)(1-\mu) > 0$, thus $t(\beta-2)-(3-2\beta)(1-\mu) < 0$, then $\frac{dD_{\{i,j\}}^{YY}}{dc_B} < 0$.

$\frac{d\pi_i^{YY}}{dc_B} = \frac{d\pi_j^{YY}}{dc_B} = \frac{(2-\beta)(\beta-1)(2-t^3\mu^2+2\tau^2)}{(t^2(\beta+2)^2-(\mu+1)^2)^2}$; It is very easy to verify $(2-\beta)(\beta-1)(2-t^3\mu^2+2\tau^2) < 0$, We know $\beta < 1, t < 1$ *and* $\mu < 1$, *thus* $\beta - 1 < 0$ *and* $t^3\mu^2 < 1$, *thus* $2 - t^3\mu^2 + 2\tau^2 > 0$, then, $\frac{d\pi_{\{i,j\}}^{YY}}{dc_B} < 0$.

Proof of Lemma 1.

Since $\theta_i^{NN} = \theta_j^{NN} = \frac{\tau(\alpha+(\beta-1)(w+c_p)}{t(2-\beta)+(\mu-1)\tau^2}$ and $\theta_i^{YN} = \frac{(\alpha+(w+c_p)(\beta-1))B+Fc_B}{A}$; Letting $\theta_i^{YN} - \theta_i^{NN} = \frac{\beta\mu+\mu+c_B+t-t\tau^2}{\tau+t(2-2t+\beta+4\tau^2-\tau)}$; We know $t < 1$ and $\tau \le 1$, thus, $t > t\tau^2$, then, $\theta_i^{YN} > \theta_i^{NN}$.

Since $\theta_i^{NN} = \theta_j^{NN} = \frac{\tau(\alpha+(\beta-1)(w+c_p)}{t(2-\beta)+(\mu-1)\tau^2}$ and $\theta_j^{YN} = \frac{(\alpha+(w+c_p)(\beta-1))G+Hc_B}{A}$;

Letting $\theta_j^{YN} - \theta_j^{NN} = \frac{\tau^2(1-2t)+\beta c_B}{t+2t\tau^2+\mu\tau^2-t\beta-t\beta\mu(\tau+\tau^2)}$, the range of the solution depends on $1-2t$, when $t \le \frac{1}{2}$, then $\theta_j^{YN} > \theta_j^{NN}$, otherwise ,$\theta_j^{YN} < \theta_j^{NN}$.

Since $\theta_i^{YN} = \frac{(\alpha+(w+c_p)(\beta-1))B+Fc_B}{A}$ and $\theta_j^{YN} = \frac{(\alpha+(w+c_p)(\beta-1))G+Hc_B}{A}$;

Letting $\theta_i^{YN} - \theta_j^{YN} = \frac{(1+\mu)\tau^2+(2t+(\beta\mu-1)\tau^2)c_B}{A}$, Expanding the numerator of the equation we can get $\tau^2 + \mu\tau^2 + 2tc_B - \tau^2 c_B + \beta\mu\tau^2 c_B$, it can be seen that when $t > \tau$, $\theta_i^{YN} > \theta_j^{YN}$, otherwise, $\theta_i^{YN} < \theta_j^{YN}$.

Since $\pi_i^{NN} = \pi_j^{NN} = \frac{t(\alpha+(\beta-1)(w+c_p))^2(2t-\tau^2)}{t(2-\beta)+(\mu-1)\tau^2}$ and

$\pi_i^{YN} = \frac{t(2t-1)((\alpha+(w+c_p)(\beta-1))(\beta^2-4)(t(2+\beta)-(1+\mu)\tau^2)+c_B(\beta^2-4)(t(\beta^2-2)+(1-\beta\mu)\tau^2))^2}{2A^2(\beta^2-4)^2}$,

Letting $\pi_i^{YN} - \pi_i^{NN} = \frac{4-t(10-2\beta^2)+\tau+2\beta\mu(\tau-1)\tau+2\mu^2\tau^3((-4+\beta^2)^2+\mu\tau^2-t\beta)}{2A^2(\beta^2-4)^2-t(2-\beta)+(\mu-1)\tau^2}$, we can set c_1^a to be the solution of $\pi_i^{YN} - \pi_i^{NN}$, According to the numerator of π_i^{YN} we can get

$c_B = \frac{w-\alpha-w\beta+c_p-\beta c_p}{1-\beta}, c_B < c_1^b =$

$\frac{t(2t-1)((\alpha+(w+c_p)(\beta-1))(\beta^2-4)(t(2+\beta)-(1+\mu)\tau^2)+c_B(\beta^2-4)(t(\beta^2-2)+(1-\beta\mu)\tau^2))^2}{2A^2(\beta^2-4)^2}$, when $0 \leq$

$c_B < c_1^a$, $\pi_i^{YN} > \pi_{\{i,j\}}^{NN}$; when $c_1^a \leq c_B < c_1^b$,$\pi_i^{YN} \leq \pi_{\{i,j\}}^{NN}$; The equilibrium is $c_B = c_1^a$.

We know $\pi_j^{YN} = \frac{t(2t-\tau^2)((\alpha+(w+c_p)(\beta-1))(\beta^2-4)(1-t(2+\beta)+\mu\tau)+c_B(\beta^2-4)((t-1)\beta+\mu\tau))^2}{2A^2(\beta^2-4)^2}$

and $\pi_i^{NN} = \pi_j^{NN} = \frac{t(\alpha+(\beta-1)(w+c_p))^2(2t-\tau^2)}{t(2-\beta)+(\mu-1)\tau^2}$.

Letting $\pi_j^{YN} - \pi_{i,j}^{NN} = \frac{2t(4-\beta^2)+4(1-\beta t-\mu\tau)-\beta^2(4-\beta t-\mu\tau)+(4-\beta^2)(\beta t-\mu\tau)c_B}{2t-t\beta-2t\tau(1-2t\tau)+2t^2\beta^2-(4-\beta^2)^2+\tau^3}$,

we can set c_1^c to be the solution of $\pi_j^{YN} - \pi_{i,j}^{NN}$, According to the numerator of π_j^{YN},

we can get $c_B = \frac{(2t^2+t\tau^2)(+2t-\beta t-\mu\tau-1)(w+w\beta+\alpha+c_p-\beta c_p)}{t\beta+\beta-\mu\tau}, c_B < c_1^d =$

$\frac{t(2t-\tau^2)((\alpha+(w+c_p)(\beta-1))(\beta^2-4)(1-t(2+\beta)+\mu\tau)+c_B(\beta^2-4)((t-1)\beta+\mu\tau))^2}{2A^2(\beta^2-4)^2}$, when $0 \leq c_B <$

c_1^c, $\pi_j^{YN} > \pi_{\{i,j\}}^{NN}$; When $c_1^c \leq c_B < c_1^d$, $\pi_j^{YN} < \pi_{\{i,j\}}^{NN}$; The equilibrium is $c_B = c_1^c$.

We known $\pi_i^{YN} =$

$\frac{t(2t-1)((\alpha+(w+c_p)(\beta-1))(\beta^2-4)(t(2+\beta)-(1+\mu)\tau^2)+c_B(\beta^2-4)(t(\beta^2-2)+(1-\beta\mu)\tau^2))^2}{2A^2(\beta^2-4)^2}$ and $\pi_j^{YN} =$

$\frac{t(2t-\tau^2)((\alpha+(w+c_p)(\beta-1))(\beta^2-4)(1-t(2+\beta)+\mu\tau)+c_B(\beta^2-4)((t-1)\beta+\mu\tau))^2}{2A^2(\beta^2-4)^2}$;

Letting $\pi_i^{YN} - \pi_j^{YN} = \frac{1+\tau^2-\beta\mu\tau^2+t(\beta^2-2)c_B(\beta^2-4)-(\beta+t\beta-\mu\tau)c_B(4+\beta^2)}{2A^2(\beta^2-4)^2}$, We can set c_1^e to be the solution of $\pi_i^{YN} - \pi_j^{YN}$, according to the numerator of π_i^{YN},we can get $c_B =$

$\frac{w-\alpha-w\beta+c_p-\beta c_p}{1-\beta}, c_B < c_1^b =$

$\frac{t(2t-1)((\alpha+(w+c_p)(\beta-1))(\beta^2-4)(t(2+\beta)-(1+\mu)\tau^2)+c_B(\beta^2-4)(t(\beta^2-2)+(1-\beta\mu)\tau^2))^2}{2A^2(\beta^2-4)^2}$, when $0 \leq$

$c_B < c_1^e, \pi_i^{YN} > \pi_j^{YN}$; when $c_1^e \leq c_B < c_1^b$, $\pi_i^{YN} < \pi_j^{YN}$; The equilibrium is $c_B = c_1^e$.

Proof of Lemma 2.

Comparing the amount of information disclosure and agent profit in YY, YN and NN scenarios:

Since $\theta_i^{YY} = \theta_j^{YY}$, $\theta_i^{NN} = \theta_j^{NN}$, We only need to prove the relationship between $\theta_{\{i,j\}}^{YY}$, $\theta_{\{i,j\}}^{NN}$, θ_i^{YN} and θ_j^{YN}.

We have known $\theta_i^{YY} = \frac{(w+c_p+c_B)(1-\beta)-\alpha}{1+(2-\beta)t-\mu}$ and $\theta_i^{YN} = \frac{(\alpha+(w+c_p)(\beta-1))B+Fc_B}{A}$;

Letting $\theta_i^{YY}-\theta_i^{YN}=\frac{(\beta^2-1)\mu\tau^2 c_B(w+c_B)(w+\beta+w\beta-1)c_p}{1-3\mathrm{t}\tau^2+t(4t+\beta)-\mu-\tau+\mathrm{t}\mu^2\tau^3}$, we know $\beta\le 1$, thus, we have $\beta^2-1<0$, then, $\theta_i^{YY}<\theta_i^{YN}$.

We have known $\theta_j^{YY}=\theta_i^{YY}=\frac{(w+c_p+c_B)(1-\beta)-\alpha}{1+(2-\beta)t-\mu}$ and $\theta_j^{YN}=\frac{(\alpha+(w+c_p)(\beta-1))G+Hc_B}{A}$;

Letting $\theta_j^{YY}-\theta_j^{YN}=\frac{(w(\beta-1)c_p)(2\mathrm{t}\tau+\tau^2+\beta\tau c_B)}{1+4t^2-2\mathrm{t}\tau^2+\beta\mathrm{t}+\tau^3-\mu(1+\mu\tau^3)}$, we know $\beta\le 1$, thus $\beta-1<0$ and $\left(w(\beta-1)c_p\right)<0$, then, $\theta_j^{YY}<\theta_j^{YN}$.

Since $\theta_i^{YY}=\theta_j^{YY}=\frac{(w+c_p+c_B)(1-\beta)-\alpha}{1+(2-\beta)t-\mu}$ and $\theta_i^{NN}=\theta_j^{NN}=\frac{\tau(\alpha+(\beta-1)(\mathrm{w}+c_p)}{t(2-\beta)+(\mu-1)\tau^2}$;

Letting $\theta_{i,j}^{YY}-\theta_{i,j}^{NN}=\frac{w(1-\beta)-\tau w(1-\beta)+(1-\beta)c_B+\alpha+(1-\beta)(1-\alpha\tau)c_p}{1-t(\beta-2)-\mu-(1+\mu)\tau^2-t(2+\beta)}$; We know $\beta\le 1$ and $\tau<1$, thus, we have $w(1-\beta)>\tau w(1-\beta)$ and $\alpha>(1-\beta)(1-\alpha\tau)c_p$, then, $\theta_i^{YY}>\theta_i^{NN}$.

Since $\pi_j^{YY}=\pi_i^{YY}==\frac{(\alpha+\left(w+c_p+c_B\right)(\beta-1))^2t^3(2-\beta)A-t(1-\mu)\mathrm{B}}{2((\mu-1)^2-t^2(\beta-2)^2)^2}$, according to the numerator of $\pi_{\{i,j\}}^{YY}$, we can get $c_B=\frac{2t^3w-\alpha-2t^3w\beta-2\mathrm{t}\tau^2+\mathrm{t}\beta^2\mu^2\tau^2+\tau^3+2t^3c_p-2t^3\beta c_p}{2t^3(-1+\beta)}$, $c_B<\mathrm{c}_1^{\mathrm{g}}=\frac{(\alpha+\left(w+c_p+c_B\right)(\beta-1))^2t^3(2-\beta)A-t(1-\mu)\mathrm{B}}{2((\mu-1)^2-t^2(\beta-2)^2)^2}$, thus, there is a common upper threshold $\mathrm{c}_1^{\mathrm{g}}$ in scenario YY.

$\pi_i^{YY}=\pi_j^{YY}=\frac{(\alpha+\left(w+c_p+c_B\right)(\beta-1))^2t^3(2-\beta)A-t(1-\mu)\mathrm{B}}{2((\mu-1)^2-t^2(\beta-2)^2)^2}$ and

$\pi_i^{YN}=\frac{t(2t-1)((\alpha+\left(w+c_p\right)(\beta-1))(\beta^2-4)(t(2+\beta)-(1+\mu)\tau^2)+c_B(\beta^2-4)(t(\beta^2-2)+(1-\beta\mu)\tau^2))^2}{2A^2(\beta^2-4)^2}$

Letting $\pi_i^{YY}-\pi_i^{YN}=\frac{t(1+\mu)-c_B(t^2(1-2t)\beta^2(4-\beta^2)-(1-\beta)t^3(2+\beta))-\tau^2(1-\mu^2\tau)}{(t^2(2-\beta)^2-(1-\mu)^2)^2+2-\tau^2(1-\mu^2\tau)-4t^3(2+t-\tau)}$, We can set $\mathrm{c}_1^{\mathrm{f}}$ to be the solution of $\pi_i^{YY}-\pi_i^{YN}$. When $0\le c_B<\mathrm{c}_1^{\mathrm{f}}$, then, $\pi_i^{YY}>\pi_i^{YN}$; When $\mathrm{c}_1^{\mathrm{f}}<c_B<\mathrm{c}_1^{\mathrm{g}}$, then, $\pi_i^{YY}\le\pi_i^{YN}$; The equilibrium is $c_B=\mathrm{c}_1^{\mathrm{f}}$.

Since $\pi_j^{YN}=\frac{t(2t-\tau^2)((\alpha+\left(w+c_p\right)(\beta-1))(\beta^2-4)(1-t(2+\beta)+\mu\tau)+c_B(\beta^2-4)((t-1)\beta+\mu\tau))^2}{2A^2(\beta^2-4)^2}$ and $\pi_i^{YY}=\pi_j^{YY}=\frac{(\alpha+\left(w+c_p+c_B\right)(\beta-1))^2t^3(2-\beta)A-t(1-\mu)\mathrm{B}}{2((\mu-1)^2-t^2(\beta-2)^2)^2}$;

Letting $\pi_j^{YY}-\pi_j^{YN}=\frac{\left(w+c_p+c_B\right)t^3(2-t)+\tau t+\mu^2\tau^2-\tau^2(w-c_p)-c_B(t\beta+\beta)}{(4t\tau+2\mu^2\tau^2)^2(\beta^2-4)^2}$, We can set $\mathrm{c}_1^{\mathrm{h}}$ to be the solution of $\pi_j^{YY}-\pi_j^{YN}$; When $0\le c_B<\mathrm{c}_1^{\mathrm{h}}$, then, $\pi_j^{YY}>\pi_j^{YN}$; When $\mathrm{c}_1^{\mathrm{h}}<c_B<\mathrm{c}_1^{\mathrm{g}}$, $\pi_j^{YY}\le\pi_j^{YN}$;The equilibrium is $c_B=\mathrm{c}_1^{\mathrm{h}}$.

We known $\pi_i^{YY}=\pi_j^{YY}=\frac{(\alpha+\left(w+c_p+c_B\right)(\beta-1))^2t^3(2-\beta)A-t(1-\mu)\mathrm{B}}{2((\mu-1)^2-t^2(\beta-2)^2)^2}$ and $\pi_i^{NN}=\pi_j^{NN}=$

$\frac{t(\alpha+(\beta-1)(w+c_p))^2(2t-\tau^2)}{t(2-\beta)+(\mu-1)\tau^2}$

Letting $\pi_{i,j}^{YY}-\pi_{i,j}^{NN}=\frac{t\left(t(2-\beta)-(1-\mu)-\tau\left(t\tau^2-\beta\mu\tau-(2-\beta\mu\tau)\tau\right)\right)}{2((1-\mu)^2+t(2-\beta)^2)^2-(1-\mu)\tau^2}$, We can set c_1^k to be the solution of $\pi_i^{YY}-\pi_i^{NN}$. When $0\le c_B<c_1^k$, then, $\pi_{i,j}^{YY}>\pi_{i,j}^{NN}$; When $c_1^k<c_B<c_1^g$, then $\pi_{i,j}^{YY}\le\pi_{i,j}^{NN}$; The equilibrium is $c_B=c_1^k$.

Proof of extensions

Proof of cost-sharing contracts

The profit functions of agents and platforms are as follows

$$\pi_i^{CYY}=\left(p_i-w-c_p-\eta c_B\right)(\alpha-p_i+\beta p_j+\theta_i-\mu\theta_j)-\frac{1}{2}t{\theta_i}^2$$

$$\pi_j^{CYY}=\left(p_j-w-c_p-\eta c_B\right)(\alpha-p_j+\beta p_i+\theta_j-\mu\theta_i)-\frac{1}{2}t{\theta_j}^2$$

$$\pi_p^{CYY}=\left(c_p-(1-\eta)c_B\right).D_T^{YY}-\frac{1}{2}t{\theta_T}^2,\ \ T=i+j$$

The corresponding first partial derivatives are:

$$\frac{\partial\pi_i^{CYY}}{\partial p_i}=w+\alpha+\eta c_B+c_p-2p_i+p\beta_j+\theta_i-\mu\theta_j$$

$$\frac{\partial\pi_j^{CYY}}{\partial p_j}=w+\alpha+\eta c_B+c_p-2p_j+p\beta_i+\theta_j-\mu\theta_i$$

$$\frac{\partial\pi_i^{CYY}}{\partial\theta_i}=(p_i-w-\eta c_B-c_p)-t\theta_i$$

$$\frac{\partial\pi_j^{CYY}}{\partial\theta_j}=(p_j-w-\eta c_B-c_p)-t\theta_j$$

Letting $\frac{\partial\pi_i^{CYY}}{\partial p_i}$=0, $\frac{\partial\pi_j^{CYY}}{\partial p_j}=0$, $\frac{\partial\pi_i^{CYY}}{\partial\theta_i}$=0, $\frac{\partial\pi_j^{CYY}}{\partial\theta_j}$=0, the optimal sales price and the corresponding optimal price information disclosure quantity are obtained as follows.

$$\theta_i^{CYY}=\theta_j^{CYY}=\frac{(w+c_p+\eta c_B)(1-\beta)-\alpha}{1+(\beta-2)t-\mu}$$

$$p_i^{CYY}=p_j^{CYY}=\frac{t\alpha-\left(w+c_p+\eta c_B\right)(\mu-1-t)}{1-t(\beta-2)-\mu}$$

$$\pi_i^{CYY}=\pi_j^{CYY}=\frac{\left(\alpha+\left(w+c_p+\eta c_B\right)(\beta-1)\right)^2t^3(2-\beta)A-t(1-\mu)B}{2((\mu-1)^2-t^2(\beta-2)^2)^2}$$

$$\pi_p^{CYY}=\frac{2t\left(\left(w+c_p+\eta c_B\right)(1-\beta)-\alpha\right)\left(QW\left(\alpha+\left(\omega+c_p\right)(\beta-1)+(\beta-1)\eta c_B\right)-JV^2\left((\eta-1)c_B+c_p\right)\right)}{QV^2K}$$

We set up some useful expressions as follows：

$A=2t(2-\beta)+(2-2\beta+4(\beta-1)\mu)$;

$B=(1-\mu)+2t(5-3\mu+\beta(2\mu-3));$
$J=t(\beta-2)-(2\beta-3)(\mu-1);$
$Q=t(\beta-2)-\mu+1;$
$K=1-t(\beta-2)-\mu;$
$V=1+(\beta-2)t-\mu;$

Since $\pi_i^{CYY}=\pi_j^{CYY}=\frac{\left(\alpha+(w+c_p+\eta c_B)(\beta-1)\right)^2 t^3(2-\beta)A-t(1-\mu)B}{2((\mu-1)^2-t^2(\beta-2)^2)^2}$,We have $\eta<\frac{\alpha+(\omega+c_p)(\beta-1)}{(1-\beta)c_B}$.

Proof of Dishonest Punishment

In scenario DYN, the profits of both agents are:

$$\pi_i^{DYN}=(p_i-w-c_p-c_B).(\alpha-p_i+\beta p_j+\tau(\theta_i-\mu\theta_j)-\frac{1}{2}t{\theta_i}^2-(\varphi F)p_i$$

$$\pi_j^{DYN}=(p_j-w-c_p-c_B).(\alpha-p_j+\beta p_i+\tau(\theta_j-\mu\theta_i))-\frac{1}{2}t{\theta_j}^2$$

The corresponding first partial derivatives are:

$$\frac{\partial\pi_i^{DYN}}{\partial p_i}=(w+\alpha+c_B+c_p-2p_i+p\beta_j+\tau(\theta_i-\mu\theta_j))-\varphi Fp_i$$

$$\frac{\partial\pi_j^{DYN}}{\partial p_j}=w+\alpha+c_B+c_p-2p_j+p\beta_i+\tau(\theta_j-\mu\theta_i)$$

$$\frac{\partial\pi_i^{DYN}}{\partial\theta_i}=(p_i-w-c_B-c_p)-t\theta_i-\varphi Fp_i$$

$$\frac{\partial\pi_j^{DYN}}{\partial\theta_j}=(p_j-w-c_B-c_p)-t\theta_j$$

Letting $\frac{\partial\pi_i^{DYN}}{\partial p_i}$=0, $\frac{\partial\pi_j^{DYN}}{\partial p_j}=0$, $\frac{\partial\pi_i^{DYN}}{\partial\theta_i}$=0, $\frac{\partial\pi_j^{DYN}}{\partial\theta_j}$=0, the optimal sales price and the corresponding optimal price information disclosure quantity are obtained as follows.

$$\theta_i^{DYN}=\frac{\left(\alpha-(\omega-c_p)(\beta+1)\right)B-G(\varphi F-c_B)}{A}$$

$$\theta_j^{DYN}=\frac{\tau(\left(\alpha-(\omega-c_p)(\beta+1)\right)H-J(\varphi F-c_B))}{A}$$

$$p_i^{DYN}=\frac{((\omega+c_p)+\alpha)}{(2-\beta)}+\frac{(\alpha+(\omega+c_p)(\beta-1))(B\beta\mu\tau-H(\beta-2\mu)\tau^2-2B)+(c_B+\varphi F)(2A-2G+G\beta\mu\tau+J(\beta-2\mu)\tau^2)}{A(4-\beta^2)}$$

$p_j^{DYN} = \frac{((\omega+c_p)+\alpha)}{(2-\beta)} +$

$\frac{(\alpha+(\omega+c_p)(\beta-1))(B\beta-2B\mu\tau+H(2-\beta\mu)\tau^2)+(c_B+\varphi F)(-A\beta+G\beta-2G\mu\tau+J(-2+\beta\mu)\tau^2)}{A(\beta^2-4)}$

Substituting the optimal sales price and optimal price information into the target's revenue function, we can get the maximum profit of the two agents:

$\pi_i^{DYN} =$

$\frac{t(2t-1)((\alpha+(\omega+c_p)(-1+\beta))(\beta^2-4)(t(2+\beta)-(1+\mu)\tau^2)+((\varphi*F+c_B)(-4+\beta^2)(t(-2+\beta^2)+(1-\beta\mu)\tau^2)))^2}{2A^2(\beta^2-4)^2}$

$\pi_j^{DYN} = \frac{t(2t-\tau^2)((\alpha+(\omega+c_p)(-1+\beta))(\beta^2-4)(1-t(2+\beta)+\mu\tau)+((\varphi*F+c_B)(\beta^2-4)((t-1)\beta+\mu\tau)))^2}{2A^2(\beta^2-4)^2}$

We set up some useful expressions as follows：

$A = t^2(\beta^2-4) - t\beta\mu\tau(1+\tau) + \tau^2(\mu^2\tau-1) + 2t(1+\tau^2);$

$B = t(2+\beta) - (1+\mu)\tau^2;$

$G = t(\beta^2-2) + (1-\beta\mu)\tau^2;$

$H = t(2+\beta) - \mu\tau - 1;$

$J = \beta - t\beta - \mu\tau;$

$L = -2G + A(-2+\beta^2) + G*\beta*\mu*\tau + J(\beta-2\mu)\tau^2;$

$M = 2A - 2G + G*\beta*\mu*\tau + J(\beta-2\mu)\tau^2;$

$V = (B*\beta*\mu*\tau - H(\beta-2\mu)\tau^2 - 2B);$

$U = -B*\beta + 2B*\mu*\tau + H(-2+\beta\mu)\tau^2;$

$W = -A*\beta + G*\beta - 2G*\mu*\tau + J(-2+\beta*\mu)\tau^2;$

In scenario DYY, the profits of both agents are:

$$\pi_i^{DYY} = (p_i - w - c_p - c_B).(\alpha - p_i + \beta p_j + \theta_i - \mu\theta_j) - \frac{1}{2}t\theta_i{}^2 - (\varphi F)p_i$$

$$\pi_j^{DYY} = (p_j - w - c_p - c_B).(\alpha - p_j + \beta p_i + \theta_j - \mu\theta_i) - \frac{1}{2}t\theta_j{}^2 - (\varphi F)p_j$$

The corresponding first partial derivatives are:

$$\frac{\partial\pi_i^{DYY}}{\partial p_i} = (w + \alpha + c_B + c_p - 2p_i + p\beta_j + \theta_i - \mu\theta_j) - \varphi F p_i$$

$$\frac{\partial\pi_j^{DYY}}{\partial p_j} = (w + \alpha + c_B + c_p - 2p_j + p\beta_i + (\theta_j - \mu\theta_i) - \varphi F p_j$$

$$\frac{\partial\pi_i^{DYY}}{\partial\theta_i} = (p_i - w - \varphi F - c_B - c_p) - t\theta_i - \varphi F p_i$$

$$\frac{\partial\pi_j^{DYY}}{\partial\theta_j} = (p_j - w - \varphi F - c_B - c_p) - t\theta_j\text{-}\varphi F p_j$$

Letting $\frac{\partial\pi_i^{DYY}}{\partial p_i}$=0， $\frac{\partial\pi_j^{DYY}}{\partial p_j} = 0$， $\frac{\partial\pi_i^{DYY}}{\partial\theta_i}$=0， $\frac{\partial\pi_j^{DYY}}{\partial\theta_j}$=0, the optimal sales price and the corresponding optimal price information disclosure quantity are obtained as follows：

$$\theta_i^{DYY} = \theta_j^{DYY} = \frac{\alpha + (\omega + c_p + c_B + \varphi F)(\beta - 1)}{1 - 2t + t\beta - \mu}$$

$$p_i^{DYY} = p_j^{DYY} = \frac{t((\omega+c_p+c_B+\varphi F)+\alpha)}{t(2-\beta)+\mu-1} + \frac{(2\alpha+(\omega+c_p+c_B+\varphi F)\beta)(\mu-1)}{(\beta-2)(1+t(-2+\beta)-\mu)}$$

Substituting the optimal sales price and optimal price information into the target's revenue function, we can get the maximum profit of the two agents:

$$\pi_i^{DYY} = \pi_j^{DYY} = \frac{(\alpha+(\omega+c_p+c_B+\varphi F)(\beta-1))^2(t(2-\beta)(2t(2-\beta)-10+\beta+8\mu)+8(1-\mu)^2)}{2(\beta-2)^2(1+t(\beta-2)-\mu)^2}$$

Proof of Variable Blockchain Costs

In the VYN scenario, the profit function of the two agents is as follows:

$$\pi_i^{VYN} = \left(p_i - w - c_p - c_B - (c_{vB}.\theta_i)\right).D_i^{VYN} - \frac{1}{2}t\theta_i^{\,2}$$

$$\pi_j^{VYN} = \left(p_j - w - c_p\right).D_j^{VYN} - \frac{1}{2}t\theta_j^{\,2}$$

The corresponding first partial derivatives are:

$$\frac{\partial \pi_i^{VYN}}{\partial p_i} = w + \alpha + c_B + c_p - 2p_i + \beta p_j + \theta_i + c_{vB}\theta_i - \tau\mu\theta_j$$

$$\frac{\partial \pi_j^{VYN}}{\partial p_j} = w + \alpha + c_p - 2p_j + p\beta_i + \tau(\theta_j - \mu\theta_i)$$

$$\frac{\partial \pi_i^{VYN}}{\partial \theta_i} = p_i - w - c_B - c_p - t\theta_i - c_{vB}\theta_i - c_{vB}\left(\alpha - p_i + \beta p_j + \theta_i - \tau\mu\theta_j\right)$$

$$\frac{\partial \pi_j^{VYN}}{\partial \theta_j} = (p_j - w - c_p) - t\theta_j$$

Letting $\frac{\partial \pi_i^{VYN}}{\partial p_i}=0$, $\frac{\partial \pi_j^{VYN}}{\partial p_j} = 0$, $\frac{\partial \pi_i^{VYN}}{\partial \theta_i}=0$, $\frac{\partial \pi_j^{VYN}}{\partial \theta_j}=0$, the optimal sales price and the corresponding optimal price information disclosure quantity are obtained as follows.

$$\theta_i^{VYN} = \frac{(\alpha + (\omega + c_p)(\beta - 1))B + F(c_B + c_{vB})}{A}$$

$$\theta_j^{VYN} = \frac{(\alpha + (\omega + c_p)(\beta - 1))G + H(c_B + c_{vB})}{A}$$

$$p_i^{VYN} = \frac{((\omega+c_p)+\alpha)}{(2-\beta)} + \frac{(\alpha+(\omega+c_p)(\beta-1))(B(\beta\mu\tau-2)+G\tau(2\mu-\beta))+(\mu\tau(2H+F\beta)-2(A+F)-H\beta\tau)(c_B+c_{vB})}{A(\beta^2-4)}$$

$$p_j^{VYN} = \frac{((\omega+c_p)+\alpha)}{(2-\beta)} + \frac{(\alpha+(\omega+c_p)(\beta-1))(G(\beta\mu-2)\tau-B(\beta-2\mu\tau))-((A+F)\beta+2H\tau-(2F+H\beta)\mu\tau)(c_B+c_{vB})}{A(\beta^2-4)}$$

$$\pi_i^{VYN} =$$

$$\frac{t(2t-1)((\alpha+(\omega+c_p)(\beta-1))(\beta^2-4)(t(2+\beta)-(1+\mu)\tau^2)+(c_B+c_{\mathrm{vB}})(\beta^2-4)(t(\beta^2-2)+(1-\beta\mu)\tau^2))^2}{2A^2(\beta^2-4)^2}$$

$$\pi_j^{VYN} = \frac{t(2t-\tau^2)((\alpha+(\omega+c_p)(\beta-1))(\beta^2-4)(1-t(2+\beta)+\mu\tau)+(c_B+c_{\mathrm{vB}})(\beta^2-4)((t-1)\beta+\mu\tau))^2}{2A^2(\beta^2-4)^2}$$

In the scenario VYY , the profit function of the two agents is as follows:

$$\pi_i^{VYY} = \left(p_i - w - c_p - c_B - (c_{vB}\theta_i)\right).D_i^{VYN} - \frac{1}{2}t\theta_i{}^2$$

$$\pi_j^{VYY} = \left(p_i - w - c_p - c_B - (c_{vB}\theta_j)\right).D_j^{VYN} - \frac{1}{2}t\theta_j{}^2$$

The corresponding first partial derivatives are:

$$\frac{\partial \pi_i^{VYY}}{\partial p_i} = w + \alpha + c_B + c_p - 2p_i + \beta p_j + \theta_i + c_{vB}\theta_i - \tau\mu\theta_j$$

$$\frac{\partial \pi_j^{VYY}}{\partial p_j} = w + \alpha + c_B + c_p - 2p_j + \beta p_i + \theta_j + c_{vB}\theta_j - \tau\mu\theta_i$$

$$\frac{\partial \pi_i^{VYY}}{\partial \theta_i} = p_i - w - c_B - c_p - t\theta_i - c_{vB}\theta_i - c_{vB}\left(\alpha - p_i + \beta p_j + \theta_i - \tau\mu\theta_j\right)$$

$$\frac{\partial \pi_j^{VYY}}{\partial \theta_j} = p_j - w - c_B - c_p - t\theta_j - c_{vB}\theta_j - c_{vB}\left(\alpha - p_j + \beta p_i + \theta_j - \tau\mu\theta_i\right)$$

Letting $\frac{\partial \pi_i^{VYY}}{\partial p_i}=0$, $\frac{\partial \pi_j^{VYY}}{\partial p_j} = 0$, $\frac{\partial \pi_i^{VYY}}{\partial \theta_i}=0$, $\frac{\partial \pi_j^{VYY}}{\partial \theta_j}=0$, the optimal sales price and the corresponding optimal price information disclosure quantity are obtained as follows.

$$\theta_i^{VYY} = \theta_j^{VYY} = \frac{(\omega + c_p + c_B + c_{\mathrm{vB}})(1-\beta) - \alpha}{1 + (\beta - 2)t - \mu}$$

$$p_i^{VYY} = p_j^{VYY} = \frac{t\alpha-(\omega+c_p+c_B+c_{\mathrm{vB}})(\mu-1-t)}{1-t(\beta-2)-\mu}$$

$$\pi_i^{VYY} = \pi_j^{VYY} = \frac{(\alpha + (\omega + c_p + c_B + c_{\mathrm{vB}})(\beta - 1))^2 t^3 (2-\beta)A - t(1-\mu)B}{2((\mu-1)^2 - t^2(\beta-2)^2)^2}$$
